# Ten principles from evolutionary ecology essential for effective marine conservation

**DOI:** 10.1002/ece3.2012

**Published:** 2016-02-28

**Authors:** Holly K. Kindsvater, Marc Mangel, John D. Reynolds, Nicholas K. Dulvy

**Affiliations:** ^1^ Earth to Ocean Research GroupDepartment of Biological SciencesSimon Fraser UniversityBurnabyBritish ColumbiaV5A 1S6Canada; ^2^ Center for Stock Assessment ResearchUniversity of CaliforniaSanta CruzCalifornia95064; ^3^ Department of BiologyUniversity of BergenBergen5020Norway

**Keywords:** Conservation, demography, extinction risk, fish, life‐history theory, management, reference points, sustainability

## Abstract

Sustainably managing marine species is crucial for the future health of the human population. Yet there are diverse perspectives concerning which species can be exploited sustainably, and how best to do so. Motivated by recent debates in the published literature over marine conservation challenges, we review ten principles connecting life‐history traits, population growth rate, and density‐dependent population regulation. We introduce a framework for categorizing life histories, POSE (Precocial–Opportunistic–Survivor–Episodic), which illustrates how a species’ life‐history traits determine a population's compensatory capacity. We show why considering the evolutionary context that has shaped life histories is crucial to sustainable management. We then review recent work that connects our framework to specific opportunities where the life‐history traits of marine species can be used to improve current conservation practices.

## Introduction

Preventing extinction and maintaining healthy marine ecosystems are common goals of fishery managers and conservation biologists, yet there is little consensus as to which populations or species are at greatest risk of extinction, and which are candidates for sustainable management. For example, two recent meta‐analyses of fish population dynamics suggest that species with fast growth and early maturity are likely to collapse from fishing pressure and environmental factors (Essington et al. 2015; Pinsky and Byler 2015). By contrast, conventional wisdom, synthesis and meta‐analysis suggest that late‐maturing species with slow life histories have an elevated risk of overexploitation and extinction (Reynolds et al. 2005; Juan‐Jordá et al. 2015). Other examples include the debate surrounding the importance of old females to future generations (Hixon et al. 2014; Shelton et al. 2015), and whether spatial closures or fisheries management is the most effective tool of conservation biologists (Edgar et al. 2014; MacNeil et al. 2015; Shiffman and Hammerschlag in press).

Naturally the truth falls somewhere in the middle of each of these debates. Here, we show that understanding the evolutionary connection between individual life‐history traits and population dynamics can relieve the tension over each of these topics. We review recent work that clarifies the ecological and evolutionary factors contributing to sustainability. We have organized our points into ten principles that bring together insights from evolutionary ecology, fisheries science, and conservation biology, and have included mathematical and empirical analyses to support them (Appendices S1–S3).

Density‐dependent regulation is central to concepts of sustainability and management, because it determines population stability and fisheries yield. Thus, the central theme of our review is that selection on species’ life‐history traits is intertwined with the strength of density‐dependent regulation of populations. We will show how density‐dependent regulation can be quantified with life‐history‐based metrics that have been developed for use in fisheries. We present a framework, POSE, that explicitly connects characteristic life histories – Precocial, Opportunistic, Survivor, and Episodic – to their compensatory capacity. The compensatory capacity determines a population's ability to withstand various types of mortality, including fishing. Finally, we review recent work highlighting that there is no single solution for managing human activities to conserve and ensure sustainability of a population. Appropriate tools depend on a species’ life history, the threat, and the set of conservation and management values.

## Population Growth and Density Dependence can be Modeled in Several Ways

One of the universal “laws” in ecology is that population dynamics are determined by a few fundamental properties of species and their environment (Lawton 1999). The trajectory of a population depends on the per capita birth rate *b* and death rate *d*, such that without density dependence, population size *N* changes according to *rN*, where *r* = *b* – *d* (Table 1 row A). Any per capita change in population growth rate with increasing density is known as density dependence. It is revealed in the relationship between *r* and *N,* which is usually negative.

**Table 1:** Common models and metrics used to quantify population growth

	Model name	Equation	Criteria for persistence	Interpretation of units	Biological description
A	Discrete population growth	$N\left(t+1\right)=N\left(t\right)+rN\left(t\right)$ where *r* = *b* – *d*	*b* > *d*	*b* is the per capita production of progeny per time *t*;* d* is the fraction of current individuals dying per time *t*	Population growth with nonoverlapping generations and no density dependence
B	Population growth	$\frac{dN}{dt}=Ne^{rt}$	*r* ≥ 0	*r* is the intrinsic rate of population growth per time *t*	Continuous population growth without density dependence
C	Logistic population growth	$\frac{dN}{dt}=rN\left(1-\frac{N}{K}\right)$	*r* ≥ 0	*r* is the rate of population growth per time *t*;* K* is the number of individuals in the steady state population	Continuous population growth with density dependence
D	Stock‐recruitment relationship	$\frac{dN}{dt}=\frac{\alpha N}{1+\beta N}-MN$	*α* > *M* at low density; $\frac{\alpha }{M}>1$ at high density	*α* is the per capita production of new individuals; *β* is 1/individuals; *M* is deaths per time *t*	Continuous population dynamics with density‐dependent survival of juveniles (see Appendix S1)
E	Spawning Potential Ratio (SPR)	Age‐ and size‐structured model with density dependence (Appendix S2)	Low SPR means fishing has eroded lifetime egg production (LEP)	Proportional change in offspring production at a given level of fishing mortality per time (*F*)	Index of recruitment per spawner in a fished stock vs. unfished population
F	Steepness in stock‐recruitment relationship	$h=\frac{0.2(N_{F=0})}{N_{F=0}}$	*α* > *M* at low density; as *α* increases *h* → 1	*h* is a proportional change in offspring production when a population is at 20% of its unfished level (*N*_*F*=0_). This could also be in units of biomass	Arises from population dynamics with density dependence (see Appendix S1)
G	Life tables	*R*_0_ = ∑_*a*_ *l*(*a*)*m*(*a*)	*R*_0_ ≥ 1	*R*_0_ is the lifetime production of daughters	Lifetime fitness in age‐structured population; also known as spawners per spawner
H	Euler – Lotka equation	1 = ∑_*a*_ *e* ^−*ra*^ *l*(*a*)*m*(*a*)	*r* ≥ 0	*r* is the instantaneous rate of age‐structured population growth	*r* is the age‐structured population growth rate; it is greatest at small population sizes; does not incorporate population density

The simplest model of population growth rate with density dependence is the logistic model (Table 1, row C), in which *r* increases and then decreases linearly as population size *N* increases (Appendix S1). The increase in *r* near zero is a result of positive density dependence. In this model, negative density dependence in the per capita death rate is determined by predation (top‐down regulation) or resource limitation (bottom‐up regulation), or some combination of both (Munch et al. 2005). The per capita birth rate is potentially limited at high densities of adults if resources or space are limited for juveniles or adults. Accordingly, fisheries models of population dynamics assume that density dependence in the birth rate of new individuals captures the biology of both adult crowding and juvenile competition (Myers 2002). While the focus of most fisheries models is a statistical description of patterns in data, we discuss the biological mechanisms that generate these patterns, keeping in mind that both density‐independent and density‐dependent mechanisms determine population growth rate and trajectory. These mechanisms are intertwined with species’ biology, including physiology and life history (Hutchings 2000).

## Carrying Capacity is Just One of Many Possible Steady States Determined by the Environment and Biology of a Species

**Figure 1:**
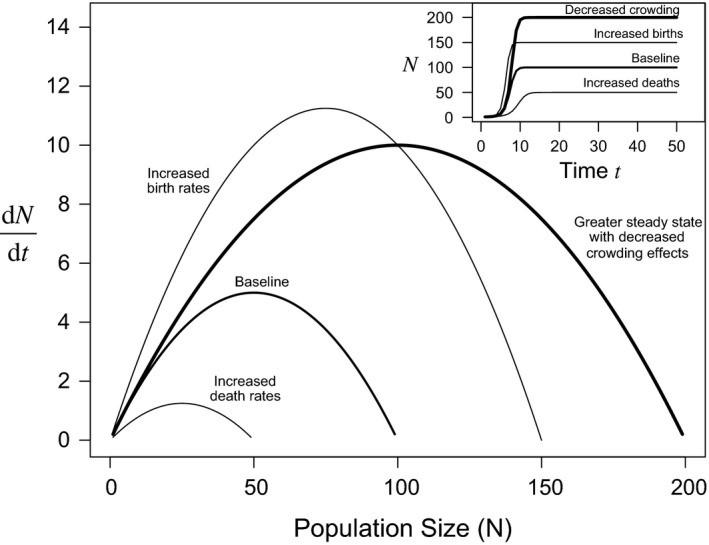
Population growth rates and steady states in the logistic population growth model. **Main panel**: Changes in birth or death rates, as well as the effect of crowding on births or deaths, affect logistic population growth and the steady state population size (Appendix S1). Notice that the effect of crowding changes only the steady state (*K)*; population growth at low population sizes is identical for both bold lines. **Inset**: the population dynamics through time for each population (line) represented in the main panel. This example is a continuous logistic model (Eq. S1.6).

The carrying capacity is the “ceiling” population size beyond which populations cannot be stable, represented in the logistic model by the parameter *K* (Table 1 Row C). The name “carrying capacity” implies the environment is like a jug that can carry a maximum quantity of water; once full, additional water will spill over the rim and be lost. However, it is not widely appreciated that in the logistic model *K* is actually a function of birth and death rates, as well as the mechanisms determining how these rates change with density (Appendix S1). This means that in the logistic family of models, including fisheries recruitment models and age‐structured models, a change in life‐history traits could change the maximum potential population size (Fig. 1), depending on the mechanism by which crowding affects birth rates or survival. Thus, while the concept of a population's “carrying capacity” has permeated the ecological literature, maximum abundance is not fixed. Instead, life‐history traits (*r* = *b* – *d*) and physiology interact with the environment to determine population abundance (or biomass) at the steady state, or the equilibrium population size (Box 1). In nature, populations fluctuate around their steady state for many reasons, including natural variability (environmental stochasticity) as well as anthropogenic effects caused by fishing or habitat loss (Box 1). Recent meta‐analyses of marine fishes have demonstrated that the life‐history traits of a population or species determine its ability to cope with this environmental variability, as well as to compensate for increased death rates due to human activity (Bjørkvoll et al. 2012; Juan‐Jordá et al. 2015). Therefore, understanding life history–environment interactions is integral to sustainability.

Box 1 **The steady state**

The logistic model (Table 1 rows C and D) illustrates a very useful concept, the steady state, where population growth rate $\frac{dN}{dt}=0.$ Of course, populations in a steady state deviate from the average growth rate of 0, but they are expected to be stable over a long period of time despite these short‐term fluctuations. This means that if perturbed, the population will eventually return to this state if the perturbation or disturbance ends. We use the term *steady state* in place of stable state or equilibrium because we want to emphasize that populations can be stable at many different sizes (see figure).

All that is necessary for a population to be in a steady state is that births equal deaths; in age‐structured populations, the proportion of the population in each age class must be constant over time. The rate of return to a steady state will depend on species’ life‐history traits, particularly generation time (the average age of adults). Populations that have been perturbed from historical levels can still be stable indefinitely, even without recovering to their previous abundance or biomass, if an increase in per capita birth rates, or a decrease in natural mortality, compensates for increased mortality due to the perturbation. Once population growth can no longer keep up with increased mortality, the population (or species) will decline toward extinction. In the figure below, we use a model of an age‐structured population with overlapping generations; the details of this model can be found in Appendix S2.

**Box 1 Figure 1:**
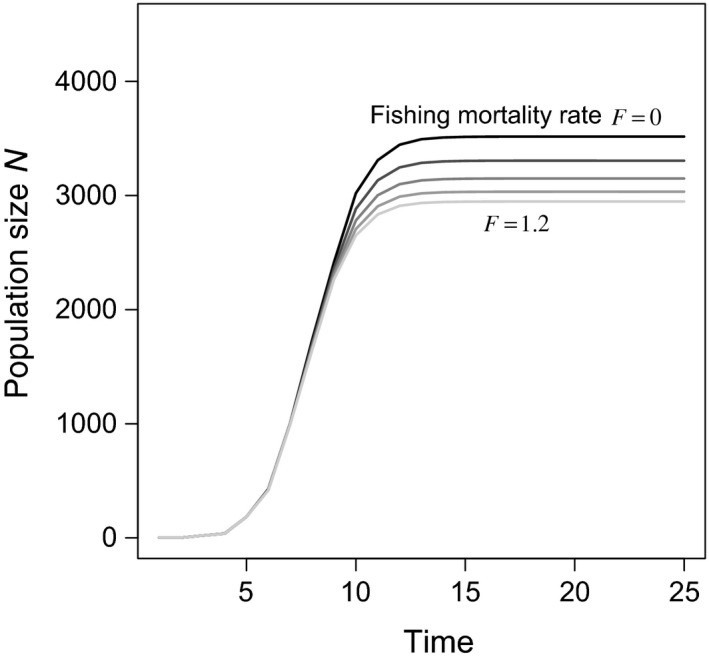
Different levels of fishing intensity *F* are represented by each line. The figure shows that multiple steady states are possible, although as fishing mortality increases, the steady state abundance decreases. Notice that the relative effect of *F* on the steady state decreases as *F* increases because *F* is a coefficient in an exponential function (Table 1; Appendix S2).

## The Compensatory Capacity of Populations Relies on Density‐Dependent Regulation

Density‐dependent regulation of population dynamics depends on the life‐history traits of a population or species (Coulson et al. 2008; Saether et al. 2013). If per capita births or juvenile survival rates increase at reduced population sizes, this increased production (*r*) can compensate for increased death rates of adults from fishing. Some species (including many fish) can also compensate by growing faster at lower densities (Gardmark et al. 2006; Lorenzen 2008). In fisheries, metrics known as reference points have been developed to quantify population or stock characteristics. These metrics indicate a population's vulnerability to overexploitation as well as its potential yield. As we will show, they can be used to determine the compensatory capacity of a population. For that reason, reference points are of use to both fisheries managers and conservation biologists.

Reference points relate some characteristic of a depleted population – such as biomass or egg production – to its baseline. In fisheries, this means that reference points are calculated for a given level of fishing effort and compared to the unfished population, resulting in a ratio. These reference metrics capture multiple changes that happen in disturbed populations, including changes in age structure, individual growth, and natural mortality and reproductive rates, without requiring a lot of assumptions about when and what type of density dependence operates. But it is important to recognize that these metrics are rooted in species’ life‐history traits, including individual birth rates, death rates, and growth rates (Clark 1991; Goodwin et al. 2006; Thorson et al. 2012; Zhou et al. 2012; Mangel et al. 2013) and will vary predictably with species’ biology. Calculating these metrics requires data on population abundance, including historical biomass, reproductive capacity, and estimates of natural mortality, as well as fishing effort (Mangel et al. 2013). Adaptive management based on reference points therefore requires regular stock assessments, something that will never happen for many of the world's exploited populations or species (Sadovy 2005).

Despite these data requirements, in some cases life‐history trait data and the size distribution of the catch can be used to calculate the reference point known as the Spawning Potential Ratio (SPR), which is the proporational egg production of a depleted population relative to its unfished egg production (Goodyear 1980; O'Farrell and Botsford 2005; Brooks and Powers 2007). For this reason, this metric has gained popularity as a metric of population sustainability for data‐limited fisheries (Brooks et al. 2010; Hordyk et al. 2014; Nadon et al. 2015). For a given level of exploitation, the SPR is a quantitative index of the compensatory capacity of a population. We next review how considering the evolution of life‐history traits can inform the compensatory capacity of populations when the data needed to calculate SPR are unavailable.

## Life‐History Traits are integral to a Population's Compensatory Capacity

**Figure 2:**
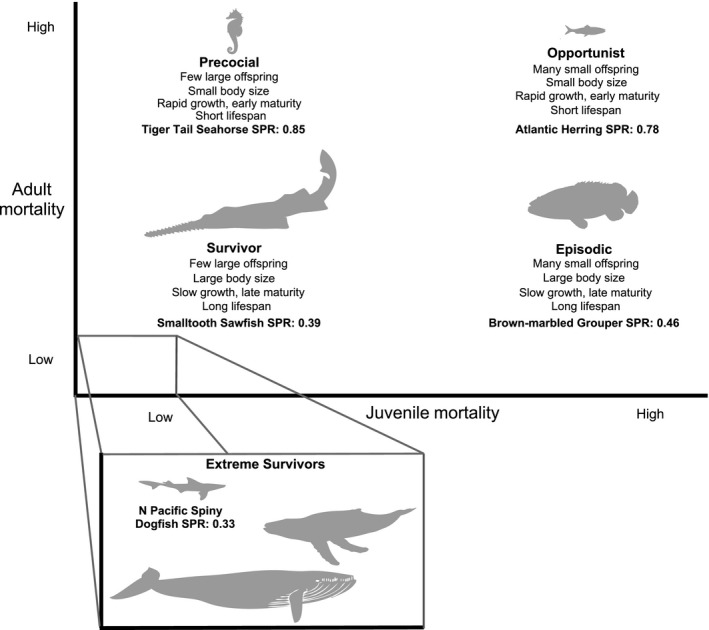
Differential mortality of juveniles and adults selects for different life histories (POSE, Precocial–Opportunistic–Survivor–Episodic), resulting in differences in compensatory capacity, quantified here for a set level of fishing mortality (*F *=* *0.2). Reproductive traits, body size, growth, age at maturity, and lifespan coevolve according to size‐independent juvenile mortality and adult mortality. We illustrate the connection between life‐history traits and compensatory capacity by calculating the Spawning Potential Ratio (SPR_F__ = 0.2_) for a fished species in each quadrant (see Appendix S2; Precocial: Tiger Tail Seahorse *Hippocampus comes*; Opportunist: Atlantic Herring *Clupea harengus*; Episodic: Brown‐marbled Grouper *Epinephelus fuscoguttatus*; Survivor: Smalltooth Sawfish *Pristis pectinata;* Extreme Survivor: North Pacific Spiny Dogfish *Squalus suckleyi*). **Inset:** Life histories with the lowest compensatory capacity, Extreme Survivors. This combination of life‐history traits characterizes species of greatest conservation concern. Illustrations are not to scale.

It is helpful to broadly categorize species based on their life‐history traits (Fig. 2) and compare their compensatory capacities. Age at maturation, body size, and offspring size and number evolve in response to selection from predation, resource availability, and environmental stochasticity (Bell 1980; Stearns 1992; Conover and Munch 2002; Walsh and Reznick 2009; Kindsvater and Otto 2014; Kindsvater et al. in review). Closely related species within the same family will be more similar to each other due to their shared evolutionary history.

In Figure 2, the vertical dimension (adult mortality rate) corresponds to the existing paradigm of a continuum between slow and fast life histories (i.e., slow life histories have low adult mortality, large body sizes, and low abundance; fast life histories have high mortality, small body sizes, and high abundance). We have extended this paradigm in the horizontal dimension (juvenile mortality rate) to explain the remaining variation in compensatory capacity. This framework builds on previous work examining the role of environmental variability in explaining life‐history variation (Winemiller and Rose 1992; Winemiller 2005; Grime and Pierce 2012). It refines prior work addressing how life‐history traits indicate species’ risk of overexploitation or extinction (Adams 1980; Purvis et al. 2000; Reynolds 2003; Reynolds et al. 2005; Hutchings et al. 2012). We focus, however, on the relative mortality risk of adults and juveniles, because selection from environmental variability acts through the mortality risk experienced by individuals. Specifically, our framework organizes life histories into four strategies: Precocial, Opportunistic, Survivor, and Episodic (POSE).

In Figure 2, we use taxonomically distant species that represent extreme life histories, but these comparisons could also be made among species in the same lineage (Cortés 2000; Juan‐Jordá et al. 2013). For each representative species, we used a size‐ and age‐structured population dynamics model, parameterized with life‐history data, to calculate the SPR for the same intensity of fishing, SPR_F_. This provides an index of the compensatory capacity of each life history (Table 1 Row E; Appendix S2). The differences in SPR for the same level of fishing mortality *F* show how anthropogenic activity interacts differently with each life‐history type; in an unperturbed population, the SPR will be 1, and in a depleted population, it will be near 0.

When adult mortality is high, selection favors earlier maturation, unless reproduction itself is the main driver of adult mortality (e.g., Kindsvater et al. in review). High background mortality or high reproductive costs tend to coevolve with small body size and short lifespans, resulting in rapid population growth rates. In Fig. 2, we consider how increasing background mortality (i.e., fishing) changes SPR_F = 0.2_. This exercise shows that while both density‐dependent and density‐independent processes regulate the population dynamics of early‐maturing species, compensatory capacity will be greatest in small species like anchovies or herring (“Opportunistic” species; in our example SPR_F = 0.2_ = 0.78) and seahorses (“Precocial” species; example SPR_F = 0.2_ = 0.85). It may be surprising that seahorses (which have parental care) are predicted to bounce back quickly from exploitation, despite their high per‐offspring investment. This is because of their early age at maturity (less than a year). Opportunistic species like herring are highly productive, and their early age at maturity allows them to capitalize on favorable environments. This also makes them susceptible to decline when environments are poor, explaining why fishing can magnify population collapses (Shelton and Mangel 2011; Essington et al. 2015).

Juvenile mortality of fish is frequently a function of chance processes, such as the encounter of predators, food, or suitable habitat during dispersal (Winemiller and Rose 1993; Mangel 2000). When juvenile survival increases rapidly with size, greater parental investment per offspring – including prolonged gestation or parental care – evolves, along with a concomitant reduction in fecundity. For example, larger offspring are advantageous when there is a size advantage in density‐dependent competition (Perez and Munch 2010; Schrader and Travis 2012; Kindsvater and Otto 2014). In this case, if adult mortality is also low, selection favors increased investment per offspring, long lifespans, and large body sizes (“Survivor” species in Fig. 2). Survivor species – including large mammals and chondrichthyans such as sawfishes – tend to have slow growth and few, relatively large offspring. They can have obligate parental care, as in whales. Selection for these traits reduces the effects of crowding on population growth rate (Travis et al. 2013). Therefore, the compensatory capacity of these species must be low (in Fig. 2, example SPR_F = 0.2_ = 0.39 or less), and they are highly likely to be threatened (Dulvy et al. 2014). In Figure 2, we show the relative placement of several extreme Survivor species on the spectrum, to illustrate that even within Survivor species, compensatory capacity varies.

Low juvenile mortality and high adult mortality are associated with large offspring and early maturity (“Precocial” species in Fig. 2), which increase the comp‐ensatory capacity of populations of these species (exam‐ple SPR_F = 0.2_ = 0.85). These traits are found in species with parental care, such as seahorses. When size‐ or density‐independent mortality of juveniles is high, selection is expected to favor large numbers of offspring instead of increased investment per offspring (Winemiller and Rose 1993). Indeed, fecundity might serve as a useful proxy for juvenile survival. Therefore, in environments where adult mortality is low, and juvenile mortality is high, species assemblages will have bet‐hedging life histories (“Episodic” species in Fig. 2). Episodic species such as groupers, Pacific rockfishes, or Atlantic Cod (*Gadus morhua*) typically have long lifespans, slow growth, and highly variable recruitment. Density‐independent environmental processes, such as unfavorable climatic regimes, can overwhelm the potential for compensation in their population dynamics, and their compensatory capacity is relatively low (example SPR_F = 0.2_ = 0.46).

With this framework in mind, we next review how life‐history traits and the Spawning Potential Ratio are related to traditional metrics of population growth *R*_0_, and how they can be used to improve population management, even where time series of abundance are scarce.

## Metrics of Individual Fitness are Useful Indicators of Population Productivity

Classic demographic models based on life‐history trait data (age‐specific birth and death rates, or life‐table data) can be used to calculate the intrinsic population growth rate *r* and the per‐generation per capita reproduction *R*_0_ using the Euler–Lotka equation (Table 1 rows G, H; Appendix S1. A population with an *R*_0_ near 1 is expected to only be replacing itself, while one with an *R*_0_ **> 1 will eventually grow to a new steady state. If *R*_0_ is less than 1, further declines are expected because individuals are not replacing themselves. *R*_0_ is very similar to the fishery index of spawners per spawner (sometimes called spawners per recruit, where in this case “recruit” is a fish that has recently become vulnerable to a fishery based on its size).

The SPR is calculated with the same data that are used to calculate *R*_0*,*_ although it is compared to a historical baseline value of *R*_0_ (i.e., without anthropogenic disturbance), and the populations are assumed to be in a steady state, rather than declining or increasing. However, this equivalence means that in data‐limited situations, demographic data used for *R*_0_ can be used to calculate the compensatory capacity of a population, as long as population size structure, age or size at maturity, and age‐ or size‐specific fecundity are known, and if there are historical reference data (Nadon et al. 2015). This may sound like a lot, but these values can be estimated by measuring individuals caught in a fishery, hunted, or otherwise removed. It is not essential to find data on abundance, recruitment, or anthropogenic mortality rates, which are much more difficult to measure.

Age‐specific survival and fecundity rates can also inform which life stages are most important for population productivity, and hence management. Reproductive value (Box 2) is a useful metric for this concept. Reproductive value represents the fitness of a female of a given age or older (i.e., current and future fitness) in a steady state population (without sex change). The various metrics of reproductive value are closely related to the lifetime egg production of females (LEP; analogous results hold for livebearers). These metrics are per‐generation estimates of population productivity (offspring produced per generation). In some cases, LEP can be easier to estimate than reproductive value, by making use of size‐specific fecundity and population size structure (e.g., estimated from fisheries catch (O'Farrell and Botsford 2005; Nadon et al. 2015). Where historical information on the relationship between size, age, and fecundity is known, calculating the LEP (or *R*_0_) of a mature female in the depleted population relative to historic female LEP (or *R*_0_) is equivalent to calculating the SPR (Table 1 rows E, G), provided the depleted population is in a steady state. The greater the ratio the more sustainable the population, because compensatory density‐dependent processes must be acting. Low LEP, relative to the historic LEP, would indicate the population has been depleted to a dangerous level (O'Farrell and Botsford 2005). This ratio allows us to judge the capacity of a species or population to withstand exploitation and recover to a target (its compensatory capacity), even if the level of depletion is unknown. It does not require any assumptions about the mechanisms of density‐dependent regulation, but rather will provide an indirect metric of the role of density dependence in the population's dynamics.

Box 2 **Reproductive value**

Demographic models, including simple life‐table models and the Euler–Lotka equation, can be used to calculate how reproductive value changes over a female's lifetime. Reproductive value is the contribution of each age class to future generations, discounted by the probability of survival to that age. It is closely related to *R*_0_, but relates these values to maternal age (or size). The relationship between reproductive value and age depends on growth, lifespan, maturation, and age‐specific survival and fecundity.

Reproductive value is confusing because it has been defined and used in several ways (Appendix S3). An early definition was simply an individual's current and future fitness at a given age, discounted by the chance of surviving to that age (Eq S3.1, Fig. S3.1a). This represents the reproductive value of an individual, given that it survives. As not all individuals survive to all ages, it is more useful to rescale this quantity as the current and future contribution of each age class relative to the total offspring production of the steady state population (Eq S3.2). The two metrics are related, but the former is a property of a long‐lived individual, the latter a property of a population.

This raises a second source of confusion about the units of reproductive value, which are often scaled for a specific purpose, for example, relative to the fitness of a juvenile. By definition, if the population is in a steady state, we know the female's contribution to future generations is one (female) offspring. This is always true unless a change in the environment changes the steady state. Thus, noticing how the units are scaled is less important than understanding how reproductive value changes with age, but scaling can be useful for comparisons among different populations or species, which have very different juvenile survival.

Calculating a female's contribution to reproductive value at each age – relative to the total production of a population –highlights which mature age classes are contributing the most to the productivity of a population (Eq. S3.3). We call this the “relative fitness” of each female. It is scaled so that lifetime fitness (the sum of fitness over all ages) is equivalent to 1. In the figure below, we plot the relative fitness at each age for four species with published estimates of mortality and reproductive rates (data and details in Appendix S3). Calculating the relative fitness of each age tells us the maternal age distribution of the juvenile population. In other words, what is the most probable age of a juvenile's mother? This information is very useful when considering how protecting different ages or life stages changes population growth rate (Fitzhugh et al. 2012). Note that for species with delayed maturity, low relative fitness as juveniles does not mean that these age classes are unimportant to population growth rate. In fact, juveniles are very important to population productivity if juvenile ages or stages have high expected future fitness relative to their current fitness. In stage‐structured models, the relative importance of each life stage (in terms of reproductive value) depends on the length of time the individual spends in it and its survival during that stage. Therefore, the importance of the juvenile stage for population productivity increases for species with late maturity, because they spend more time as juveniles. This explains why juvenile survival is more important to population growth rate in long‐lived, late‐maturing rays and sharks than in early‐maturing species (Cortés 2002; Frisk et al. 2005).

**Box 2 Figure 1:**
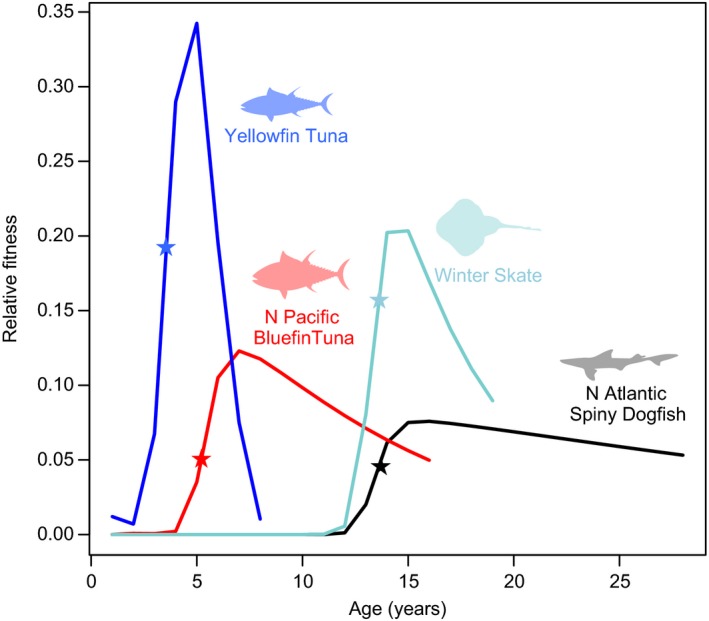
The relative fitness (the contribution of each age class to reproductive value) for four species with contrasting life histories. Mean age at maturity for each species is noted with a ★. Curves are generated from simulations based on published growth, mortality, and life‐history parameters; each curve represents the expected fitness of each age class, scaled by total births in a steady state population. For each species, mean fecundity is known – from this, the relationship between fertility and age was assumed to be proportional to body size at age. Data and supplementary graphs are in Appendix S3.

Thus, life‐history traits allow the calculation of useful proxies of fitness and compensatory capacity. But life‐history traits alone can be used to categorize species’ risk of overexploitation or potential to sustain fishing if the data needed to calculate lifetime egg production or other fitness metrics are unavailable. We next review general rules of thumb that come out of our POSE framework and our review of the connections between SPR, *R*_0_, and reproductive value.

## High Fecundity and High‐Quality Eggs are not Enough for Sustainability

That high fecundity makes fish populations resistant to overexploitation is a zombie idea, in that it has been thoroughly refuted but refuses to die (Sadovy 2001; Denney et al. 2002). Species with high fecundity may be unable to recover quickly from depleted levels, because their eggs have very low survival and their contribution to population recovery will be discounted by the time it takes to mature (Rothschild 1986). In other words, they have low reproductive value (Box 2). As a rule, changes in individual growth rates, age at maturation, and body size have a greater effect on the population dynamics of long‐lived, late‐maturing species than do egg number or egg quality (Heppell et al. 1999).

In some long‐lived species, older females produce more, higher quality eggs that have higher survival in early life (Berkeley et al. 2004; Hixon et al. 2014). However, the net contribution of these eggs to population growth rate will be low in species with late maturation and low juvenile survival. Furthermore, environmental variability is an important driver of recruitment for long‐lived, highly fecund (Episodic) species. Egg quality differences due to population age and size structure do not necessarily contribute meaningfully to long‐term population dynamics (Shelton et al. 2012, 2015; Le Bris et al. 2015).

The timing and importance of density‐dependent and density‐independent mortality will determine the importance of egg quality for population dynamics (Myers 2002; Munch et al. 2005). For example, it is possible that density‐dependent mechanisms of mortality or growth operate well after effects of egg quality on larval survival and growth have been swamped by other sources of variability, and the long‐term implications for population dynamics will be dominated by these factors. A good rule of thumb for management of long‐lived species is to protect the age classes with the greatest potential contribution to lifetime fitness (MacArthur 1960). Usually, that means females that are just starting to breed (Heppell et al. 1999) but it also includes juvenile stages in species with high per capita survival during that stage, for example, late‐maturing species like spiny dogfishes.

## Large Biomass of a Population does not Protect it from Collapse

Populations of some species can reach very high densities in productive environments. Yet this does not mean that the population is able to withstand high fishing pressure. Recent attention has revived the question of sustainability of fisheries for herring, sardines, and other forage fish (Essington et al. 2015; Pinsky and Byler 2015; Szuwalski and Hilborn 2015). Forage fish populations have supported some of the most profitable fisheries in history and have also collapsed spectacularly and repeatedly (Essington et al. 2015; Pinsky and Byler 2015). These fish are typically considered to be Opportunistic species, as they experience highly variable environments. In our deterministic calculation of SPR in Appendix S2, we showed that these species do have potentially high rates of recovery if environmental conditions are favorable for their recruitment. Yet we emphasize that density‐independent processes regulate their dynamics, so that population size and recruitment are poorly correlated, and their true compensatory capacity can be very low over short time scales. Poor environmental conditions and fishing can interact to destabilize their dynamics (Shelton and Mangel 2011). For this reason, forage fisheries can easily become overcapitalized, resulting in collapse.

Another example of species with very high biomass but low productivity are the spiny dogfishes (or Spurdog as they are known in Europe) (‘Survivor’ strategy Fig. 2). These cartilaginous fishes have extremely long gestation (nearly 2 years), low fecundity, and a long lifespan (up to 80 years in Pacific Spiny Dogfish *Squalus suckleyii*). Spiny dogfishes (*S. acanthias* and *S*. *suckleyi*) can reach very high levels of standing biomass because they have a low trophic level, feeding mainly on planktivorous fishes and invertebrates. This slow life history has led to repeated collapses of spiny dogfish fisheries, despite the fact that they are among the most abundant coastal sharks. In general, species with large standing biomass, low adult mortality, and slow growth are the slowest to recover from overexploitation (Jennings et al. 1998; Ralston 2003).

## Long Lifespans Evolved for a Reason

Long lifespans and high fecundity evolve in response to selection for persistence in highly variable (stochastic) environments (Winemiller and Rose 1993). High variability can arise from processes operating on several scales, including high uncertainty in juvenile survival due to the vagaries of oceanic currents or decades of poor juvenile survival due to unfavorable climatic conditions (Warner and Chesson 1985; Longhurst 2002; Mangel 2003). For example, Sablefish (*Anoplopoma fimbria*) – which can live for more than 90 years – can have decades between successful recruitment events (King et al. 2001). When an unfavorable environmental regime can persist for a decade or more, only long‐lived females will have the opportunity to experience a successful recruitment year (King et al. 2001). Hence, population stability of these Episodic fish depends on an occasionally successful cohort that lives a long time (McFarlane and Beamish 1992; Wright 2014).

This evolutionary perspective makes it clear that changes in age‐ and size‐structure have important consequences for the stability of populations of Episodic species, as fishing will erode the buffer against infrequent recruitment provided by old individuals (Kuparinen and Hutchings 2012). Fishing itself also leads to plastic and evolutionary changes in population demography and life history, which can decrease the population's capacity for density‐dependent compensation (Walsh et al. 2006; Swain 2010; Kuparinen and Hutchings 2012). The most important message for conservation practitioners is that truncating population age structure can be very risky for species with long natural lifespans.

## Allee Effects are Hard to Detect but Should not be Ignored

Until now, we have focused largely on the role of negative density dependence limiting populations. But it is also possible for mechanisms of positive density dependence to affect population growth rates, particularly at low population sizes (Goodyear 1980; Hutchings 2015). In other words, population growth rate increases with density or number. This pattern is known as depensation or the Allee effect. Changes in population growth rate at low population sizes can arise for many reasons. For example, sessile species such as abalone or urchins can have low fertilization success at low densities. Overexploitation of one sex, as in a size‐selective fishery on a sex‐changing fish, can also lead to sperm limitation (Alonzo and Mangel 2004; Heppell et al. 2006). Aggregating species are at risk of depensation if reproductive success depends on density (Stoner and Ray‐Culp 2000; Sadovy and Domeier 2005). Finally, predation can also lead to depensation if predator density is high enough that prey death rates increase at low prey density, or if prey are more vulnerable at low densities, which may be the case for species that cooperate for defense, such as schooling fish (Walters et al. 2000; Walters and Kitchell 2001; Dulvy et al. 2004).

The prevalence of depensation in marine populations has been widely debated (Keith and Hutchings 2012; Hilborn et al. 2014; Hutchings 2015). Detecting positive density dependence is very difficult, because the importance of stochastic processes to population dynamics increases at low population sizes. In other words, the dynamics of small populations are expected to be exceptionally noisy. For this reason, we recommend a conservative approach to estimating population recovery that leaves a buffer against low population size to prevent potential depensatory effects.

## Spatial Planning (Marine Protected Areas) Should be Informed by Life Histories

When faced with population declines and few data, many conservation practitioners have turned to Marine Protected Areas (MPAs) as a management tool, often called “spatial planning”. These areas may be designed to protect juvenile nursery habitat, or to protect species interactions with the intention of restoring ecosystem function. In some cases, MPAs are implemented with the hope they will export production to nearby areas open to exploitation (Hilborn 2004; Pelc et al. 2010). MPAs are most effective when all fishing is prohibited, enforcement is strong, and they are large, old, and isolated (Edgar et al. 2014). Even if these criteria are met, an MPA may not affect production in nearby areas, and so might not solve the problem of displaced fishing effort. Finally, while MPAs are appealing for their conceptual simplicity, designing and implementing an effective MPA is far from simple. Protected areas require continuous governance and financial investment and specifically need to account for the redistribution of displaced fishing effort, as well as the biology of the species they are designed to protect. For this reason, simple fisheries management tools (such as size limits or access limits) are essential complements to spatial protection measures.

Despite these limitations, for some species spatial protection is a highly effective method of conservation. That depends on the biology, including life history and behavior (Mangel 1998). Spatial protection is most appropriate for species that have limited home ranges, such as sessile invertebrates, or limited geographic ranges, including endemic species and species with low dispersal. Protecting habitat associated with specific life stages can be essential if natural mortality is low (e.g., sawfishes in mangroves; Morgan et al. 2015), or if reproductive individuals are clustered (e.g., during spawning aggregations or migrations (Sadovy and Domeier 2005). By the same token, MPAs are less likely to be appropriate management tools for migratory species or those with large home ranges. Finally, it is futile to protect metapopulation sinks if sources are not protected (Cooper and Mangel 1999; Burgess et al. 2014). Spatial protection will increase or maintain populations if it protects age or size classes (stages) in the locations that contribute the most to subsequent generations (e.g., those with high relative fitness; Box 2). This means that spatial management will be most effective if the individuals it protects are near maturity, if they have high survival during their time in that habitat, or if a large proportion of the population uses the area.

## Conclusion

We have emphasized the connection between life‐history traits and reference metrics for conservation and management, because the sustainability of a population depends on the species’ life history as well as environmental and anthropogenic factors. Considering where a species’ life history falls on the POSE spectrum can therefore be used to go beyond the usual cast of stock‐assessed species to diagnose vulnerability to human exploitation of data‐poor species.

We have used examples from fish and fisheries throughout this review to show that sustainable fisheries are possible even for species with extremely slow life histories (e.g., spiny dogfishes and Sablefish) and that understanding which species are likely to be sustainable can be inferred from considering the evolutionary context of their life‐history traits. In general, Precocial or Opportunistic species with high or unpredictable natural adult mortality will have greater compensatory capacity and potentially the greatest sustainable yield (Fig. 2). The clearest examples of relative sustainability come from comparisons within phylogenetic groups. For example, the life‐history traits of Yellowfin Tuna (*Thunnus albacares*) allow their populations to withstand greater fishing pressure than tuna species such as *T. orientalis* (Juan‐Jordá et al. 2015; Box 2).

Yet there is more to sustainable management than getting the biology right. A depleted population must have a positive population growth rate to recover, but the appropriate metrics of recovery are not as clear. One benchmark is recovery to a set proportion of initial population size (Brooks et al. 2010). Recovery can also imply a return to a former demographic structure (Redford et al. 2011) or ecosystem role (Hughes et al. 2007). In some cases, this means human welfare and economic interests must be weighed against the possibility of local extinction (Allison et al. 2009) and the desire to return to a baseline ecosystem state (Levin and Lubchenco 2008; Mace 2014).

The principle underlying our narrative is that management accounting for life‐history traits can lead to recovery, and eventually to resilient populations that are better able to withstand further environmental change. “Resilience” implies that a species will be able to recover from a perturbation, because of built‐in redundancy or robustness (Holling 2001; Redford et al. 2011), which here we have called compensatory capacity. In marine ecosystems, resiliency means the ability to withstand fishing pressure and habitat loss, to maintain trophic structure, to resist invasion of non‐natives, or to cope with climate change (Graham et al. 2011). However, it can also be the ability to recover from short‐term disturbances such as an oil spill. Different definitions are appropriate, depending on the scale of the problem and the goal, but the connection to life‐history traits is always present.

## Conflict of Interest

None declared.

## Supporting information


**Appendix S1.** Classic models of population dynamics in ecology and fisheries science.

**Appendix S2.** Calculation of reference points in an age‐ and size‐structured population.
**Table S2.1.** Description of the age‐structured model in the Box 1 Figure and Figure 2, including the biological processes modeled, corresponding equations, and parameter interpretations.
**Table S2.2.** Life history parameters for the analyses in Fig. 2 (main text) and Eqs. S2.1–2.5.

**Appendix S3.** Calculating reproductive value.
**Table S3.1.** Data used to calculate relative fitness of each age in Box 2.
**Fig. S3.1.** In (a) we plot *V*(*a*) over age using Eq. S3.1 and estimates of age‐specific mortality, maturity and length.
