# Supplementary material for: Ten principles from evolutionary ecology essential for effective marine conservation
Source: Ecol Evol. 2016 Feb 28;6(7):2125–38. doi: 10.1002/ece3.2012 (PMC4782246; doi:10.1002/ece3.2012)
Supplement: Supplementary file 1 — Appendix S1. Classic models of population dynamics in ecology and fisheries science. [file ECE3-6-2125-s001.pdf]

Appendix S1: Classic models of population dynamics in ecology and fisheries science

Populations do not grow indefinitely. No concept is more fundamental to ecology and evolution. Malthus hypothesized that without limiting factors a population will grow without bound; his treatise on the limits to growth motivated Darwin's notion of the struggle for existence. Any per-capita change in population growth rate with increasing density is known as density dependence. It is revealed in the relationship between r and N, which is usually negative. In a small population that is not limited by competition for food or space, r will reach its maximum.

All population models can be represented in discrete or continuous time. The difference is in the accounting of birth and death rates – discrete models average birth and death rates over a given time interval, while continuous models assume that birth rates and death rates are instantaneous (*i.e.*, they are constantly compounded; Table 1, rows B,C). The predictions of discrete- or continuous-time models will be very similar, with a few exceptions (such as deterministic chaos in discrete models for which the continuous analogue has no such behavior).

Here, we review three of the most common continuous and discrete-time population dynamics models in ecology and fisheries science (logistic, Beverton-Holt, and Ricker). For each one, we highlight where density-dependent regulation is assumed to operate.

Continuous Time Logistic Growth

Density dependence in the logistic model arises from a simple assumption about how the population growth rate changes as a function of population density. If $N(t)$ denotes the population size at time t, then in continuous time this assumption translates into the equation for exponential growth

$$\frac{dN}{dt} = rN \tag{S1.1}$$

where r is the Malthusian growth parameter (also called the intrinsic rate of increase, or simply PGR (Population Growth Rate). If $N(0)$ is the population size at $t = 0$, then the solution of Eq. S1.1 is $N(t) = N(0)e^{rt}$. The per-individual or per-capita growth rate associated with Eq. S1.1 is

$$\frac{1}{N}\frac{dN}{dt} = r \tag{S1.2}$$

so that regardless of population size, each individual produces the same average number of offspring per unit time. In recruitment models, maximum population growth rate is the slope of the population trajectory at low population sizes $\alpha - M$ where M *is* density-independent natural mortality (Table 1 row D). These models assume population growth rate declines as a function of adult population biomass and do not specify the underlying biological mechanisms responsible for the decline. There are an infinite number of ways to have per capita growth rate decline as population size increases, but there is only one linear model. Thus we introduce a constant a*,* which represents the decline in per capita growth rate as density increases. This could be due to crowding, limited resources, or increased predation at high densities. We replace Eq. S1.2 by

$$\frac{1}{N}\frac{dN}{dt} = r - aN \tag{S1.3}$$

We now rewrite Eq. S1.3 as

$$\frac{dN}{dt} = N(r - aN) \tag{S1.4}$$

If we define a new parameter K by $K = \frac{r}{a}$, then Eq. S1.4 becomes

$$\frac{dN}{dt} = rN - \frac{r}{K}N^2 \tag{S1.5}$$

Notice that K comes from a and r, and is therefore determined by the biology (r) and the interaction of the species with its with its environment (a). In light of Eq. S1.5, the growth rate or productivity of the population is parabolic, falling to 0 when $N = 0$ or $N = K$; hence the latter value is a steady state (the carrying capacity).

Factoring rN on the right hand side of Eq. S1.5 we obtain the continuous logistic model (Row C, Table 1 of the main text):

$$\frac{dN}{dt} = rN\left(1 - \frac{N}{K}\right) \tag{S1.6}$$

The solution of this equation is

$$N(t) = \frac{KN(0)}{N(0) + (K - N(0))e^{-rt}} \tag{S1.7}$$

which shows sigmoidal growth from an initial value (below K) to the steady state (K).

Discrete Time Logistic Growth

Until now we have considered differential equations that represent populations that are growing with no lag between the time an individual is born, and when it begins to reproduce. This describes the dynamics of populations with overlapping generations (and populations of bacteria, where individuals mature soon after they divide from their parent).

An alternative to differential equations – which can be hard to solve – is to use difference equations. Difference equations model reproduction in discrete time intervals. This change in accounting makes them generally easier to deal with. For example, Eq. S1.6 can be written as a difference equation

$$N(t+1) = N(t) + rN(t)\left(1 - \frac{N(t)}{K}\right) \quad \text{(S1.8)}$$

However, we cannot treat Eq. S1.8 as the precise analogue Eq. S1.6. Because there are non-overlapping generations, sometimes very weird things happen in the population dynamics of discrete-time models because of the lag between when births are calculated and when density-dependent regulation operates (see Mangel 2006, pp 40-43). This disparity between the deterministic dynamics of the discrete and continuous logistic led to the discovery of deterministic chaos (May 2002). While fascinating, this means Eq. S1.8 is not an appropriate model of the population dynamics of most species, which do not have a lag between births and deaths (largely because of overlapping generations).

Fortunately, in this case we also have the exact solution to the continuous logistic (Eq. S1.7). For this reason, we can write this solution as a difference equation without fear of chaos:

$$N(t+1) = \frac{KN(t)}{N(t) + (K - N(t))e^{-rt}} \quad \text{s} \quad \text{(S1.9)}$$

Alternative Models of Birth and Death Processes

Notice that the terms on the right-hand side of Eq. S1.5 can be interpreted as births ($b(N) = rN$) minus deaths ($d(N) = \frac{r}{K}N^2$). More generally, the growth rate of the population will be the difference between births and deaths

$$\frac{dN}{dt} = b(N) - d(N) \quad \text{(S1.10)}$$

If we assume that density dependence acts on births rather than deaths, the per capita rate of mortality will be a constant M , so that $d(N) = MN$ and Eq. S1.10 becomes

$$\frac{dN}{dt} = b(N) - MN \quad \text{(S1.11)}$$

This means the birth rate has to be a nonlinear function of population size, declining as population size increases. As above, an infinite number of choices of function are possible; we describe two here because of their common use in fisheries science.

The first is due to Beverton and Holt (1957; see also Mangel 2006) and involves two additional parameters. The first, α, characterizes maximum per capita reproduction and the second, β, the strength of density dependence. We replace the generic Eq. S1.11 by

$$\frac{dN}{dt} = \frac{\alpha N}{1+\beta N} - MN \qquad \text{(S1.12)}$$

The second is due to Ricker (1954) and also has two parameters, with the same interpretation, but a different functional form. Eq. S1.11 is replaced by

$$\frac{dN}{dt} = \alpha N e^{-\beta N} - MN \qquad \text{(S1.13)}$$

The first terms on the right hand sides of Eqs. S1.12 and S1.13 represent a Stock Recruitment Relationship (SRR) in fisheries science. In the form we have defined it, the Beverton-Holt recruitment function (Eq. S1.12) allows one to represent the asymptotic population abundance as $\frac{\alpha}{\beta}$, while the Ricker function (Eq. S1.13) has a peak and then declines as population size increases. This means the per-capita rate of production (here, α) affects the asymptote (the steady state) as well as population growth from low numbers (Fig. 1, main text).

As with the logistic equation, which has a steady state ($dN/dt = 0$) when $N = K$, Eqs. S1.12 and S1.13 have steady states, which we denote by $\bar{N}$. For Eq. S1.12 the steady state is

$$\bar{N} = \frac{1}{\beta}\left(\frac{\alpha}{M} - 1\right) \qquad \text{(S1.14)}$$

We thus conclude that the population can persist only if $\frac{\alpha}{M} > 1$.

In conservation biology, we are often interested in a population's "production", or its growth rate from low population sizes. When N is small, Eqs. S1.12 and S1.13 both are approximately αN. Furthermore if we Taylor expand Eqs. S1.12 or S1.13 to the second order we obtain the same result

$$\frac{dN}{dt} = \alpha N(1-\beta N) - MN = (\alpha - M)N - \alpha\beta N^2 \qquad \text{(S1.15)}$$

Comparing Eqs. S1.15 and S1.5 shows the similarity between logistic population growth and the Beverton-Holt and Ricker functions. Namely, $\alpha - M = r,$ and $\alpha\beta = \frac{r}{K}$; thus $\alpha, M,$ and β are different interpretations of the logistic parameters r and K.

Steepness

As a metric of productivity, Mace and Doonan (1988) introduced a new quantity h, known as steepness, which describes the birth rates of a population at 20% of its steady state level relative to the unfished birth rate. Using the notation that we have developed thus far

$$h = \frac{b(0.2\bar{N})}{b(\bar{N})} \tag{S1.17}$$

In fisheries science, models keep track of biomass instead of numbers, because fecundity (and thus productivity) is related to female mass. Therefore $\bar{N}$ in Eq. S1.17 can represent either numerical abundance or biomass at the steady state.

For the Beverton-Holt recruitment function, Mangel et al. (2010; 2013) show that steepness is

$$h = \frac{\frac{\alpha}{M}}{4 + \frac{\alpha}{M}} \tag{S1.18}$$

Thus, as α increases, h approaches 1, corresponding to very strong compensatory recruitment. This metric is related to other population reference points that are important for quantifying a population's capacity to withstand perturbations (see Table 1 in the main text and Supplement 2).

References

Beverton RJH, Holt SJ (1957) On the Dynamics of Exploited Fish Populations, Fishery Investigations Series II Volume XIX, Ministry of Agriculture, Fisheries and Food, UK

Mace PM, Doonan IJ (1988) A generalised bioeconomic simulation model for fish population dynamics. New Zealand Fishery Assessment Research Document 88/4. Fisheries Research Centre, MAFFish, POB 297, Wellington, NZ

Mangel, M (2006) *The Theoretical Biologist's Toolbox.* Cambridge University Press, New York, NY< USA

Mangel M, Brodziak JKT, DiNardo G (2010) Reproductive ecology and scientific inference of steepness: a fundamental metric of population dynamics and strategic fisheries management. Fish Fish 11**:** 89-104

Mangel M, MacCall AD, Brodziak J, Dick EJ, Forrest RE et al. (2013) A perspective on steepness, reference points, and stock assessment. Can J Fish Aq Sci 70: 930-940

May RM (2002) The Best Possible Time to Be Alive. It Must Be Beautiful. The Great Equations of Modern Science. pp 212-229 Ed.: Farmelo G. Granta Books, London, UK

Ricker WE (1954) Stock and Recruitment. J Fish Res Board Canada 11: 559-623.
