# Supplementary material for: Ten principles from evolutionary ecology essential for effective marine conservation
Source: Ecol Evol. 2016 Feb 28;6(7):2125–38. doi: 10.1002/ece3.2012 (PMC4782246; doi:10.1002/ece3.2012)

Appendix S2. Calculation of reference points in an age- and size-structured population

Population models can be extended to incorporate population age structure (the number of individuals alive at each age) or size structure (the number of individuals or biomass in each size class). Modeling age or size structure in discrete or continuous time captures the dynamics of immature and mature individuals, and allows for overlapping generations (i.e., the oldest offspring reproduce before their mother stops reproducing). This allows the use of age- or size-specific birth rates (fecundity) and death rates (mortality). Age- or size-structured models are intended to have higher fidelity to nature than the logistic model, though no model fully captures reality. The species and question being asked determine if it is sufficient to simply model biomass and assume non-overlapping generations, or whether a complex age-structured model is needed. For example, assuming generations do not overlap can be appropriate when modeling the dynamics of semelparous species such as Pacific salmon.

To illustrate the connection between life history traits and compensatory capacity, we iterated the dynamics of an age-structured population with overlapping generations as functions of both age a and time t (Mangel 2006; Mangel et al. 2010). Model equations and parameter descriptions are given in Table S2.1. From these equations we can simulate a population of a species with a given life history, and calculate the SPR in Fig. 2 (main text). We are interested in the dynamics of biomass as well as age structure, so we track both age and size in our model. This size-at-age relationship is determined by our assumptions about individual growth, which we model using the von Bertalanffy growth function (Mangel 2006). In this function the growth coefficient *k* determines how fast the fish grow when small. The asymptotic size L_∞ determines the maximum size reached by an average individual. From the relationship between age and length, we can calculate mass-at-age $W(a)$ as a simple power function determined by the exponent ω. Natural mortality M is assumed to be constant, or a function of age where possible. For simplicity, we also represent the probability of maturation $p_m(a)$ and fishing mortality $F(a)$ as functions of age a (not length or mass), but this does not change the qualitative pattern of results. Egg production $E(t)$ depends the number of individuals alive at age a, the probability they are mature at that age $p_m(a)$, and the relationship between fecundity and body mass $W(a)$. The population dynamics are regulated by Beverton-Holt density-dependence in recruitment, where the number of recruits N_0 is determined by α and β (Supplement 1). We assume recruits are the same size, L_R (in cm), and live to at most A_{max} years.

In Figs. S2.1-S2.5 we show the age-specific life history traits, mortality, recruitment, and population dynamics that result from our model for five species representing the four categories

in Fig. 2 in the main text (e.g., Atlantic Herring for Opportunistic, Marble-spotted Grouper for Episodic, Smalltooth Sawfish for Survivor, North Pacific Spiny Dogfish for Extreme Survivor, and Tiger Tail Seahorse for Precocial). We parameterized the model using life history parameters (growth, lifespan, and age at maturity; see Figs. S2.1-S2.5 a, b), and natural mortality estimates for each species (Table S2.2). Except for natural mortality M, parameters in Table S2.2 came from the primary literature and Fishbase (Table S2.2; Froese and Pauly 2015). We estimated natural mortality M from the primary literature or based on published estimates for similar species in the same category (Table S2.2; Fig. 2). We also assumed the allometric relationship between length, mass, and fecundity were similar for all species ($\omega = 0.3$, $\nu = 50$; $b = 3$; Figs S2.1-S2.5 b); we adjusted the fecundity coefficient c so that clutch sizes were close to the published values for each species (Table S2.2). For all species size at recruitment was $L_R = 4$, except for the seahorse, which we assumed recruited at size $L_R = 1$. We also assumed the dispersion of the sigmoidal maturity function was the same for all species ($q = 0.8$).

We estimated the Beverton-Holt recruitment relationship between offspring production and survival. Combining the bottom three rows of Table S2.1, in the steady state the number of recruits (age-0 individuals) is

$$\bar{N}_0 = \frac{\alpha \sum_a p_m(a) c W(a)^b) \bar{N}_0 e^{-M(a)}}{1+\beta \sum_a p_m(a) c W(a)^b \bar{N}_0 e^{-M(a)}} \quad \text{(S2.1)}$$

which we can rewrite as

$$1 = \frac{\alpha \sum_a p_m(a) c W(a)^b) e^{-M(a)}}{1+\beta \bar{N}_0 \sum_a p_m(a) c W(a)^b e^{-M(a)}} \quad \text{(S2.2)}$$

The summation in these equations use individual life history parameters to characterize total egg production in the steady state, the maximum probability that an egg survives to recruitment (α) and a metric of the strength of density dependence (β). That means both life history and population attributes enter into Eqs. S2.1 and S2.2. If we assume that α and $\bar{N}_0$ are known, then

$$\beta = \frac{1}{\bar{N}_0 \sum_a p_m(a) c W(a)^b e^{-M(a)}} \left[\alpha \sum_a p_m(a) c W(a)^b e^{-M(a)} - 1\right] \quad \text{(S2.3)}$$

In this steady state, the lifetime egg production of an individual is

$$\bar{E}_{ind} = \sum_a p_m(a) c W(a)^b e^{-M(a)} \quad \text{(S2.4)}$$

which allows us to rewrite Eq. S2.3 as

$$\beta = \frac{1}{\bar{N}_0 \bar{E}_{ind}} [\alpha \bar{E}_{ind} - 1] \quad \text{(S2.5)}$$

Consequently, if we specify the maximum probability of an egg surviving to recruitment α (i.e., steepness; Supplement 1, Mangel et al. 2010), and the number of recruits in the steady state population, we can estimate the strength of density dependence using life history traits and Eqs. 4 and 5. We estimated α with each species' annual fecundity estimate (assumed to be 1 for highly fecund species). We used $\bar{N}_0 = 100{,}000$ for each species. We checked that our simulated populations all reached a steady state within 200 years, at which time we "fished" our populations ($T_{fishing} = 200$). After a fixed time ($T_{recovery} = 400$) we stopped fishing and allowed the population to recover, which it did within 100 years ($T_{max} = 500$). All mature age classes were vulnerable to our fishery ($F(a) = 0.2$). After several generations the population reached a new steady state. We assumed the selectivity function was a knife-edge function such that fishing began at the age at maturity a_{mat}, or that it was a function of size where individuals were 0.75% of the asymptotic size L_∞. This allowed us to generate a stock-recruitment relationship for each species (Figs. S2.1-S2.5 d). By choosing such long time intervals between fishing and unfished populations, we are able to compare steady states of the fished and unfished populations. To calculate the spawning potential ratio (SPR) reported in Fig. 2 (main text), we compared lifetime egg production of individuals in fished steady state population with those in the unfished population (we did this for both types of fishery selectivity; population dynamics before, during, and after fishing are in Figs. S2.1-S2.5 e-f).

Because of these assumptions, our calculated reference point (SPR) is very simplistic, but sensitivity analyses showed that it was not strongly affected by the recruitment parameters (α, β, or L_R) or fecundity and maturity shape parameters (ω, ν, b , or q). This is because we are comparing the ratio of egg production in a lightly fished population (in this case, $F = 0.2$) relative to the unfished population. Because α and β are in both its numerator and denominator, the recruitment parameters have only a minor effect on our estimates of the ratio (until the population is very depleted). Age at maturity, growth, lifespan, and natural mortality M are much more important predictors of compensatory capacity, and therefore rates of population recovery and decline (this can be seen in Figs. S2.1-S2.5 e-f). Finally, our simulations do not include plasticity or evolution in growth rate that changes the age at maturation of each population, but future work on the interaction between life history traits, evolution or plasticity, and fishery reference points would be useful (e.g., Frisk 2010).

Table S2.1.

**Biological process**	**Equation**	**Parameter interpretation**
Von Bertalanffy growth function	$L(a+1) = L(a)e^{-k} + L_{\infty}(1 - e^{-k})$.	L_{∞} and k determine size-at-age L
Maturation probability function	$p_m(a) = \frac{1}{1 + e^{-q(a - a_{mat})}}$	a_{mat} is the age at which 50% of females mature; *q* determines the steepness ogive
Body mass (depends on $L(a)$)	$W(a) = \nu L(a)^{\omega}$	ν and ω are scale and shape parameters
Natural mortality	$M(a)$	M can be a function of age *a* or constant
Fishing mortality	$F(a) = \begin{Bmatrix} 0 & if\ a < a_{mat} \\ s(a) & if\ a \geq a_{mat} \end{Bmatrix}$	$s(a)$ is selectivity of the fishing gear, can also depend on size
Egg production of the population (all mature individuals)	$E(t) = \sum_a N(a,t)\ p_m(a)\ cW(a)^b$	Fecundity and mass depend on age *a, c* and *b*
Density dependent recruitment	$N_0(t+1) = \frac{\alpha E(t)}{1+\beta E(t)}$.	Beverton-Holt recruitment (Supplement 1).
Population dynamics through time	$N(a+1, t+1) = \begin{Bmatrix} N_0(t) & if\ a = 0 \\ N(a,t)\ e^{-(M(a)+F(a))} & if\ a > 0 \end{Bmatrix}$	*N(a,t)* is the number of individuals of age *a* alive at time *t*
Spawning Potential Ratio in the steady state population	$SPR = \frac{\sum_a^{a_{max}} W(a) p_m(a) e^{-M(a)-F(a)}}{\sum_a^{a_{max}} W(a) p_m(a) e^{-M(a)}}$	Egg production in the fished population relative to the unfished population. Note that the max ages in each population may differ

Table S2.2. Life history parameters for the analyses in Fig. 2 (main text) and Eqs. S2.1-2.5

Species	A_{max}	a_{mat}	L_∞ (cm)	k	c	M	α	β	Reference
. Atlantic Herring *Clupea harengus*	18	3	30	0.3	1	0.4	1	1×10^{-5}	Fishbase (Froese and Pauly 2015)
Brown-marbled Grouper *Epiniphelus fuscoguttatus*	40	5.5	99	0.2	10	0.1	1	1×10^{-5}	Fishbase (Froese and Pauly 2015)
Smalltooth Sawfish *Pristis pectinata*	44	9	448	0.22	0.00006	0.07	6	6×10^{-5}	Carlson and Simpfendorfer 2015
Tiger Tail Seahorse *Hippocampus comes*	5	1	16	2.9	0.007	0.3	1	1×10^{-5}	Morgan and Vincent 2013
N Pacific Spiny Dogfish *Squalus suckleyi*	80	36	90	0.07	0.00025	0.04	3	3×10^{-5}	Tribuzio and Kruse 2012

References

Carlson JK, Simpfendorfer CA (2015) Recovery potential of smalltooth sawfish, *Pristis pectinata*, in the United States determined using population viability models. Aquatic Conserv: Mar. Freshw. Ecosyst. 25: 187–200

Froese R, Pauly D (2014) FishBase. World Wide Web publication. www.fishbase.org, accessed March 2015.

Frisk MG (2010) Life History Strategies of Batoids. In: Biology of Sharks and Their Relatives 2. Physiological Adaptations, Behavior, Ecology, Conservation and Management of Sharks and Their Relatives, Eds. JC Carrier, JA Musick, MR Heithaus. CRC Press, Boca Raton, Florida.

Mangel M (2006) The Theoretical Biologist's Toolbox. Cambridge University Press, New York, NY, USA

Mangel M, Brodziak JKT, DiNardo G (2010) Reproductive ecology and scientific inference of steepness: a fundamental metric of population dynamics and strategic fisheries management. Fish Fish 11**:** 89-104

Morgan SK, Vincent ACJ (2013) Life history reference points for management of an exploited tropical seahorse. Mar Fresh Res 64: 185-200

Tribuzio CA, Kruse GH (2012) Life history characteristics of a lightly exploited stock of *Squalus suckleyi.* J Fish Biol 80: 1159-1180

Figure Captions: In Figures S2.1-2.5 we plot the age- and size-based relationships that emerge from our simulations for each species in Figure 2 (main text). Panels **a** and **b** show the relationship between body size and age and fecundity and age, which are based on the von Bertalanffy growth function. These are determined by life history data. Panel **c** shows the

Beverton-Holt recruitment function that we calculate for each species. We then simulate the population dynamics over time, with and without fishing. This allows us to determine the relationship between stock size and actual recruitment, shown in panel **d.** We show the simulated dynamics in panels **e** and **f,** with two types of fishery selectivity (based on age and size, respectively).

Figure S2.1

Atlantic Herring
Clupea harengus

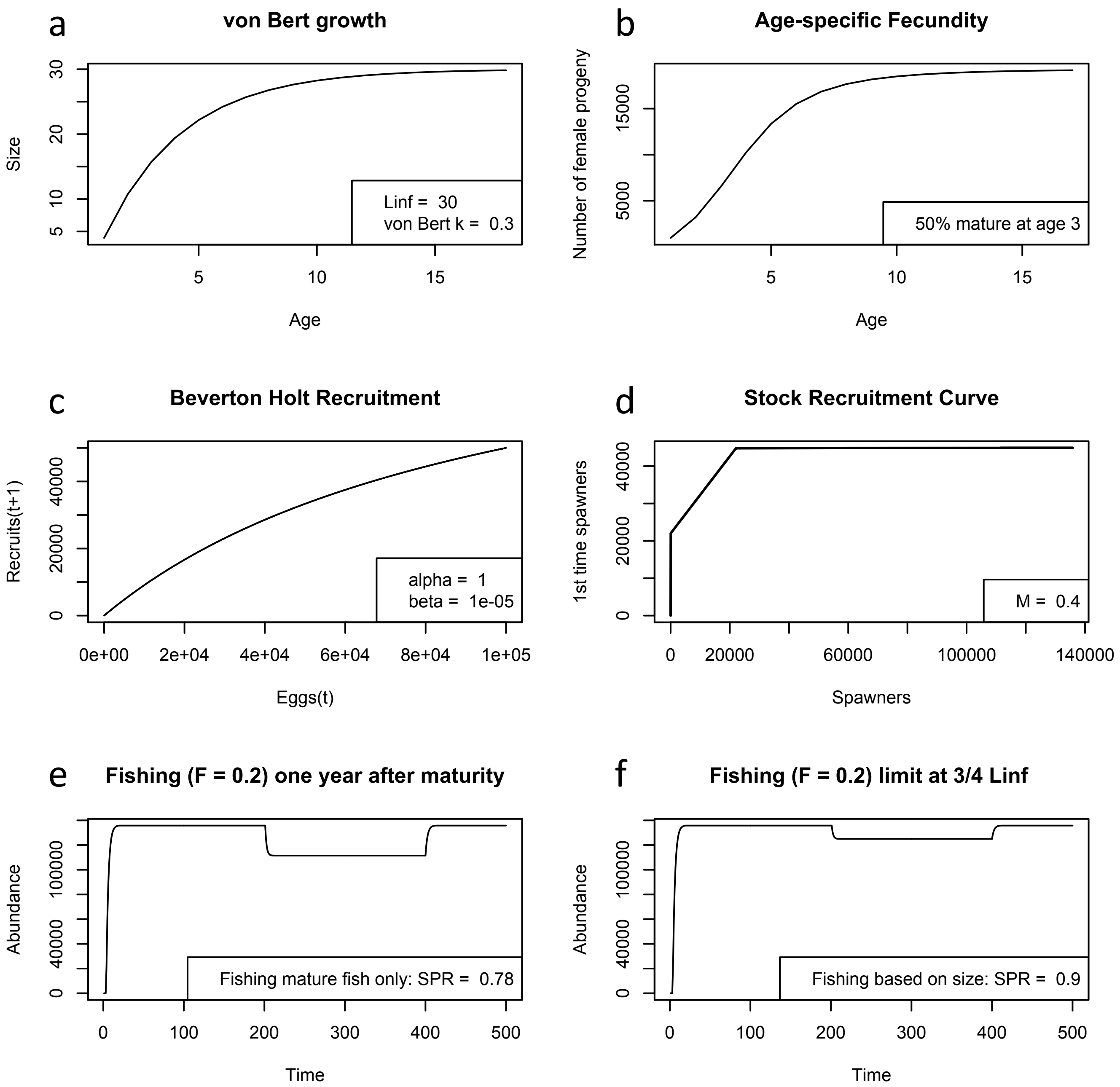

Figure S2.2

Brown-marbled Grouper
Epinephelus fuscoguttatus

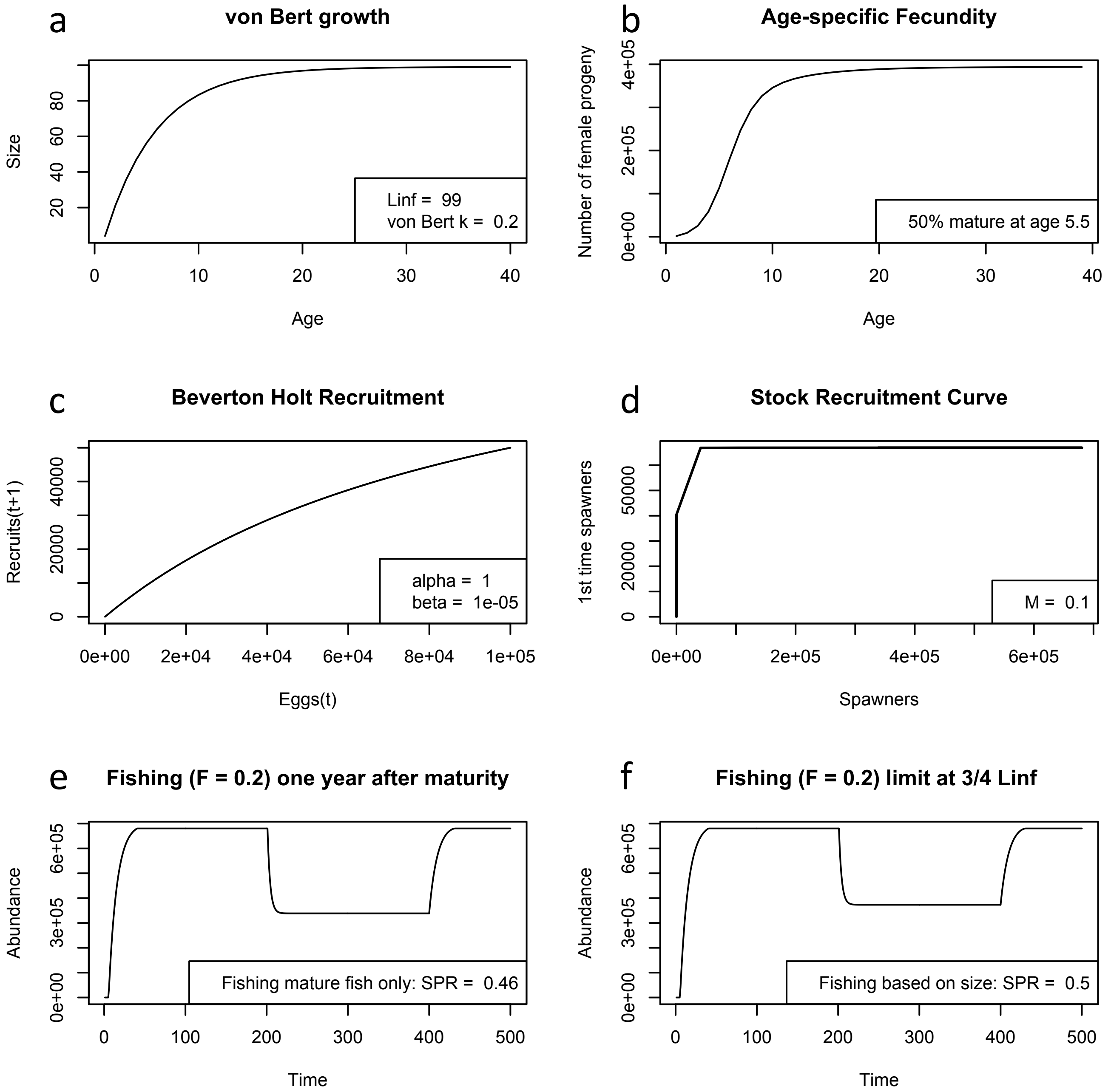

Figure S2.3 North Pacific Spiny Dogfish

Squalus suckleyi

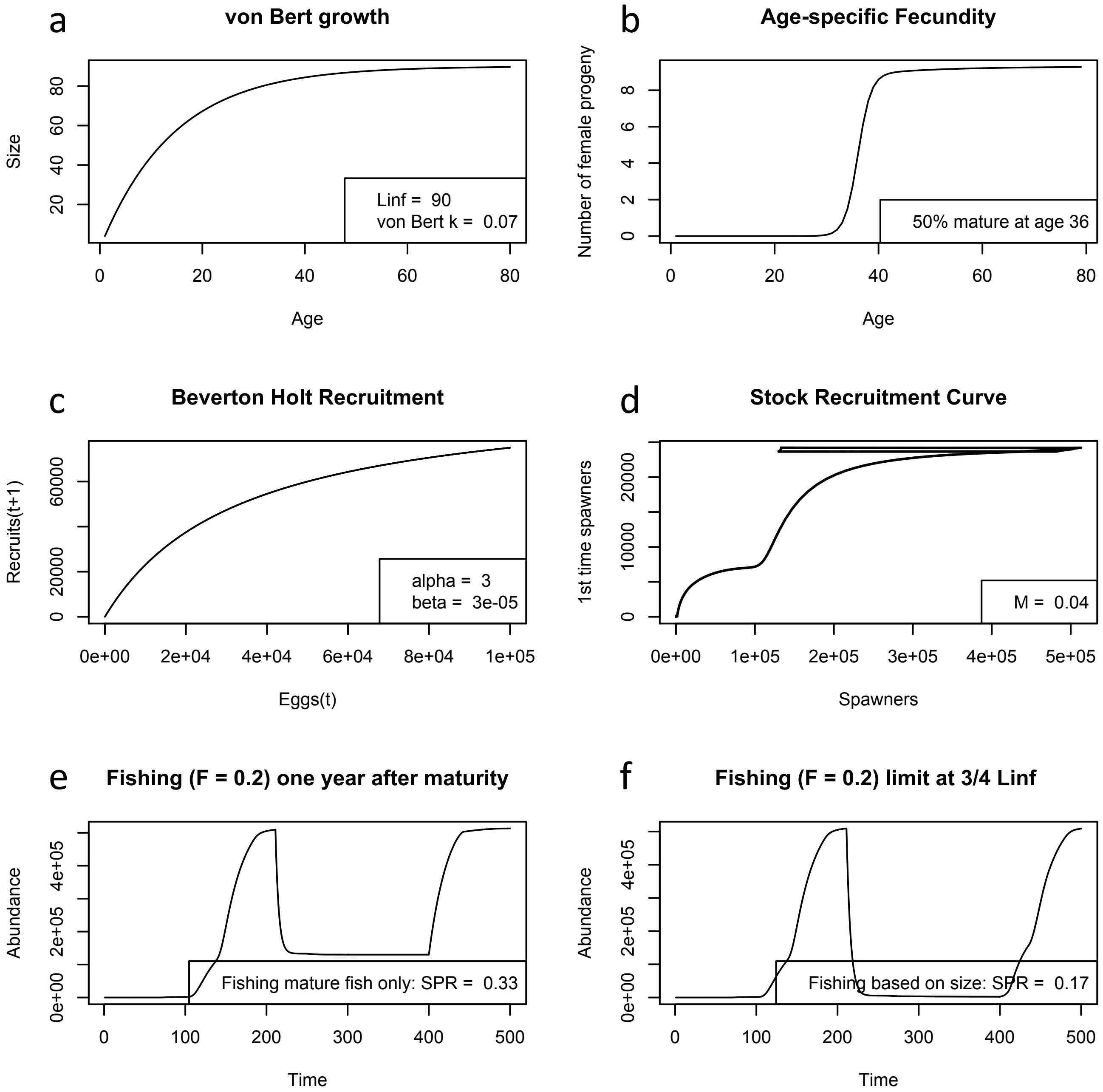

Figure S2.4

Tiger Tail Seahorse
Hippocampus comes

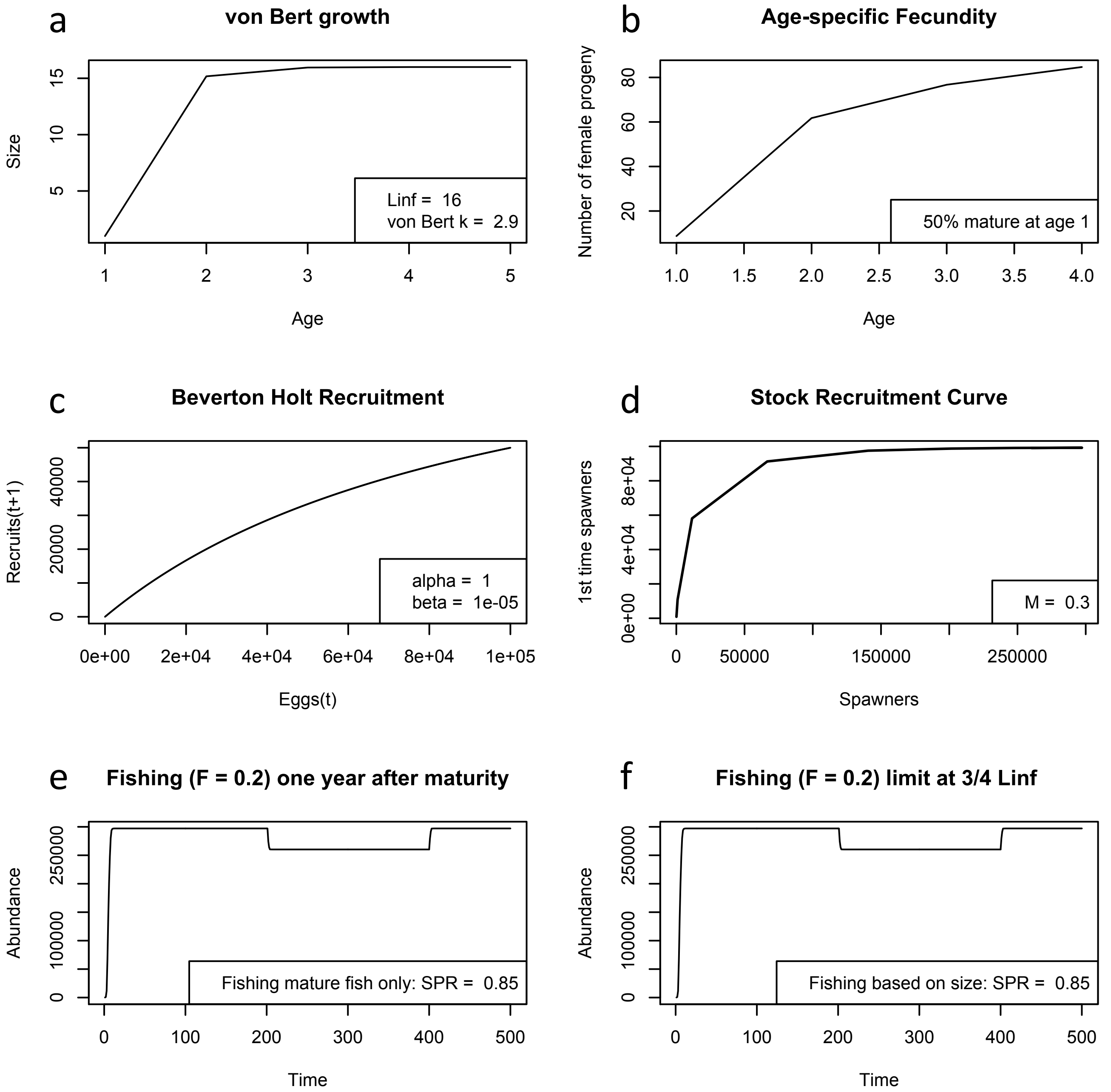

Figure S2.5

Smalltooth Sawfish
Pristis pectinata

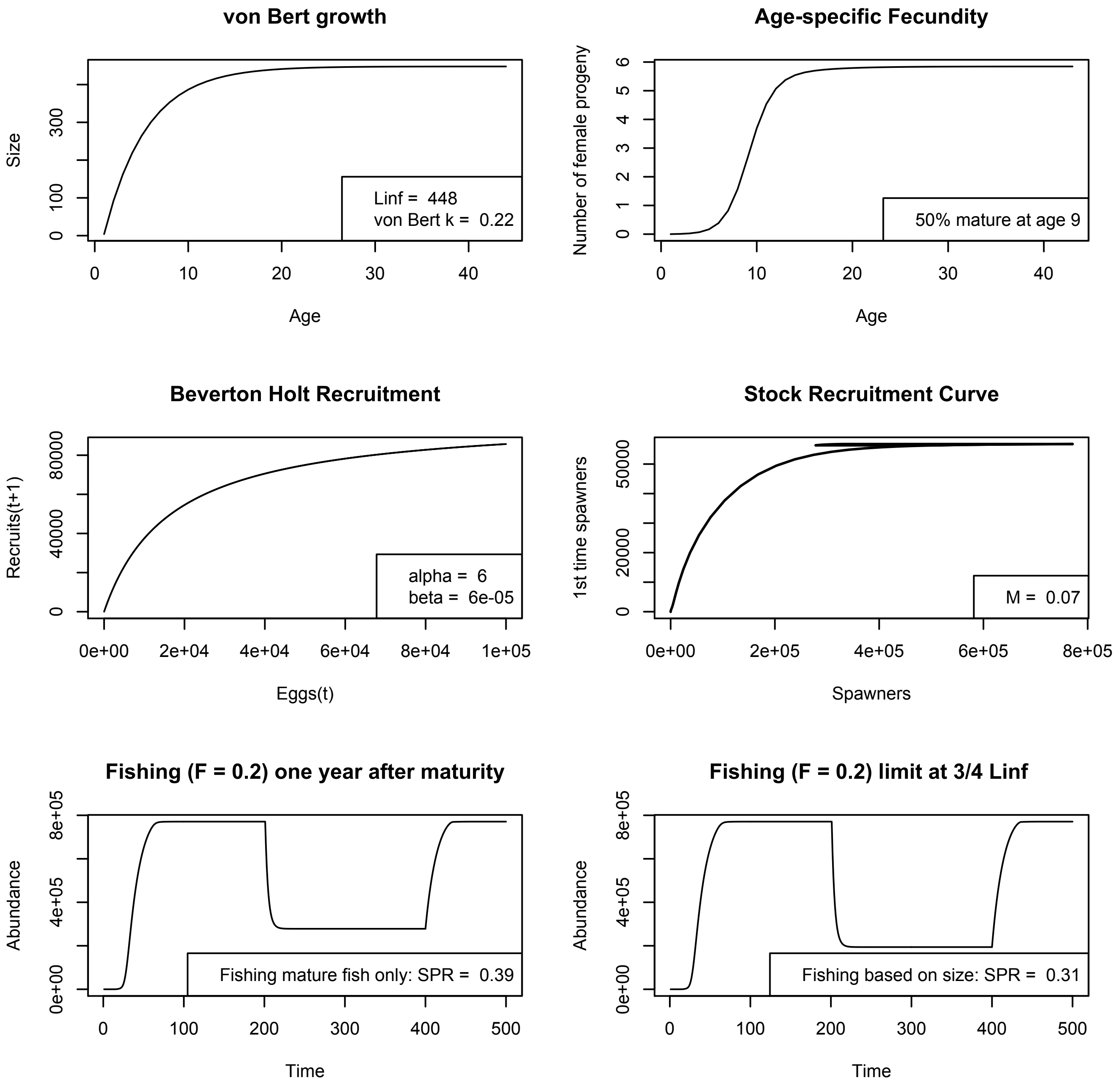

Supplement: Supplementary file 2 — Appendix S2. Calculation of reference points in an age‐ and size‐structured population. Table S2.1. Description of the age‐structured model in the Box 1 Figure and Figure 2, including the biological processes modeled, corresponding equations, and parameter interpretations. Table S2.2. Life history parameters for the analyses in Fig. 2 (main text) and Eqs. S2.1–2.5. [file ECE3-6-2125-s002.pdf]
