# Supplementary material for: Ten principles from evolutionary ecology essential for effective marine conservation
Source: Ecol Evol. 2016 Feb 28;6(7):2125–38. doi: 10.1002/ece3.2012 (PMC4782246; doi:10.1002/ece3.2012)

Appendix S3. Calculating reproductive value.

We let $S(a)$ denote survival to age *a*, $\Phi(a)$ denote fecundity at age *a*, determined by size at age and the allometry between size and fecundity, $\bar{N}(a)$ the number of individuals of age *a* in the steady state population, and $p_m(a)$ the probability that an individual of age *a* is sexually mature.

At the steady state, the growth rate of the population is 0, so that reproductive value as defined by Fisher (1930) is

$$V(a) = \frac{1}{S(a)} \sum_{a'=a}^{A} p_m(a')\Phi(a') \tag{S3.1}$$

This is the expected current and future contribution of offspring of an individual who is alive at age *a*. This metric is a bit misleading since it presumes that the individual is alive at age *a*, which may happen with vanishingly small probability (Hamilton 1996).

As a more relevant metric for conservation, we consider the contribution of individuals of age *a* or older to the total egg production of the steady state population

$$W(a) = \frac{\sum_{a'=a}^{A} \bar{N}(a') p_m(a')\Phi(a')}{\sum_{a'=1}^{A} \bar{N}(a') p_m(a')\Phi(a')} \tag{S3.2}$$

$W(a)$ is the relative contribution of each age to steady state population production. It allows us to compare the reproductive values of different ages, relative to the generation time. This definition is equivalent to the left eigenvector of an age-structured population matrix that has been scaled by juvenile reproductive value.

Finally, we can calculate relative current fitness, or the contribution of individuals exactly of age *a* to the total egg production of the steady state population

$$w(a) = \frac{\bar{N}(a) p_m(a)\Phi(a)}{\sum_{a'=1}^{A} \bar{N}(a') p_m(a')\Phi(a')} \tag{S3.3}$$

In this form, the sum of relative fitness over all ages is equivalent to lifetime fitness, again scaled to 1.

Each of these functions describes a different aspect of the relationship between expected female reproductive success and age. To illustrate this, in Fig. S3.1, we iterated the age structured model for the population dynamics of Atlantic Spiny Dogfish (*Squalus acanthias*)

populations using published estimates of life history parameters and vital rates (Marques da Silva and Ross 1993, Campana et al. 2006, Table S3.1) to show how $V(a), W(a)$, and $w(a)$ change with age (assuming fishing mortality F is zero). We used these same data to parameterize our age- and size-structured population dynamics model (described in Supplement 2). All individuals "recruited" to our population model at a minimum size L_R. We adjusted the recruitment function parameters so that the population reached a steady state (without fishing) (Fig. S3.2, bottom left panel).

For Box 2, we repeated this process for several species with age-specific life history data available (Figs. S3.3-S3.5; Table S3.1). Box 2 shows the relationship between life history traits and the relative fitness of each age class in the steady state population (using Eq. S3.3).

Table S3.1. Data used to calculate relative fitness of each age in Box 2.

Species	A_{max}	a_{mat}	$M(a)$	L_∞	k	ω	c	α	β	Reference
North Atlantic Spiny Dogfish *(Squalus acanthias)*	29	13	A_0: 0.14<br>$A_{1\text{-}5}$: 0.06<br>A_{6+}: 0.04	125	0.09	0.3	0.0004	0.74	8×10^{-8}	Marques da Silva and Ross 1993; Campana et al. 2006
Winter Skate *(Leucoraja ocellata)*	20	13	0.22<br>(age-specific estimates unavailable)	111	0.05	0.1	0.02	0.08	1×10^{-10}	Frisk 2010
North Pacific Bluefin Tuna (*Thunnus orientalis*)	17	5	A_0: 1.6<br>A_1: 0.46<br>A_2: 0.27<br>A_3: 0.20<br>A_{4+}: s0.12	320	0.1	0.3	10	0.004	1×10^{-10}	Mangel et al. 2010; Anonymous 2008
Yelllowfin Tuna (*T. albacares*)	14	3.5	A_0: 6.1<br>A_1: 1.5<br>A_2: 0.68<br>A_3: 0.44<br>A_4: 0.69<br>A_{5+}: 1.5	180	0.4	0.3	10	0.002	1×10^{-10}	Hampton 2000; Juan-Jordá et al. 2013

References

Anonymous (2008) Report of the Pacific Bluefin Tuna Working Group Workshop. International Scientific Committee for Tuna and Tuna-like Species in the North Pacific Ocean.

Fisher RA (1930) The Genetical Theory of Natural Selection. Clarendon Press, Oxford, UK.

Hamilton WD (1996) Narrow Roads of Gene Land. Vol. 1: Evolution of Social Behaviour. Oxford University Press, Oxford, UK.

Campana SE, Jones C, McFarlane GA, Myklevoll S (2006). Bomb dating and age validation using the spines of spiny

dogfish (*Squalus acanthias*). Environ Biol Fish 77: 327-336

Frisk MG (2010) Life History Strategies of Batoids. In: Biology of Sharks and Their Relatives 2. Physiological Adaptations, Behavior, Ecology, Conservation and Management of Sharks and Their Relatives, Eds. JC Carrier, JA Musick, MR Heithaus. CRC Press, Boca Raton, FL, USA

Hampton J (2000) Natural mortality rates in tropical tunas: Size really does matter. Can J Fish Aq Sci 57:1002-1010.

Juan-Jordá M J, Mosqueira I, Freire J, Dulvy NK (2013) Life in 3-D: life history strategies in tunas, mackerels and bonitos. Rev Fish Biol Fisheries 23: 135-155

Mangel M, Brodziak JKT, DiNardo G (2010). Reproductive ecology and scientific inference of steepness: a fundamental metric of population dynamics and strategic fisheries management. Fish Fish 11**:** 89-104

Marques da Silva MA, Ross MR (1993) Reproductive strategies of spiny dogfish, *Squalus acanthias*, in the NW Atlantic. ICES: C.M. G(51):18p

Figure Captions

Fig. S3.1. In (a) we plot $V(a)$ over age using Eq. S3.1 and estimates of age-specific mortality, maturity and length. We made some necessary simplifying assumptions about the relationship between fecundity and age. In (b) and (c) we used Eqs. S3.2 and S3.3 to calculate and plot the reproductive value $W(a)$ and relative fitness $w(a)$ of a female in the steady state population.

Figure S3.1: The relationship between age and different metrics of reproductive value and fitness

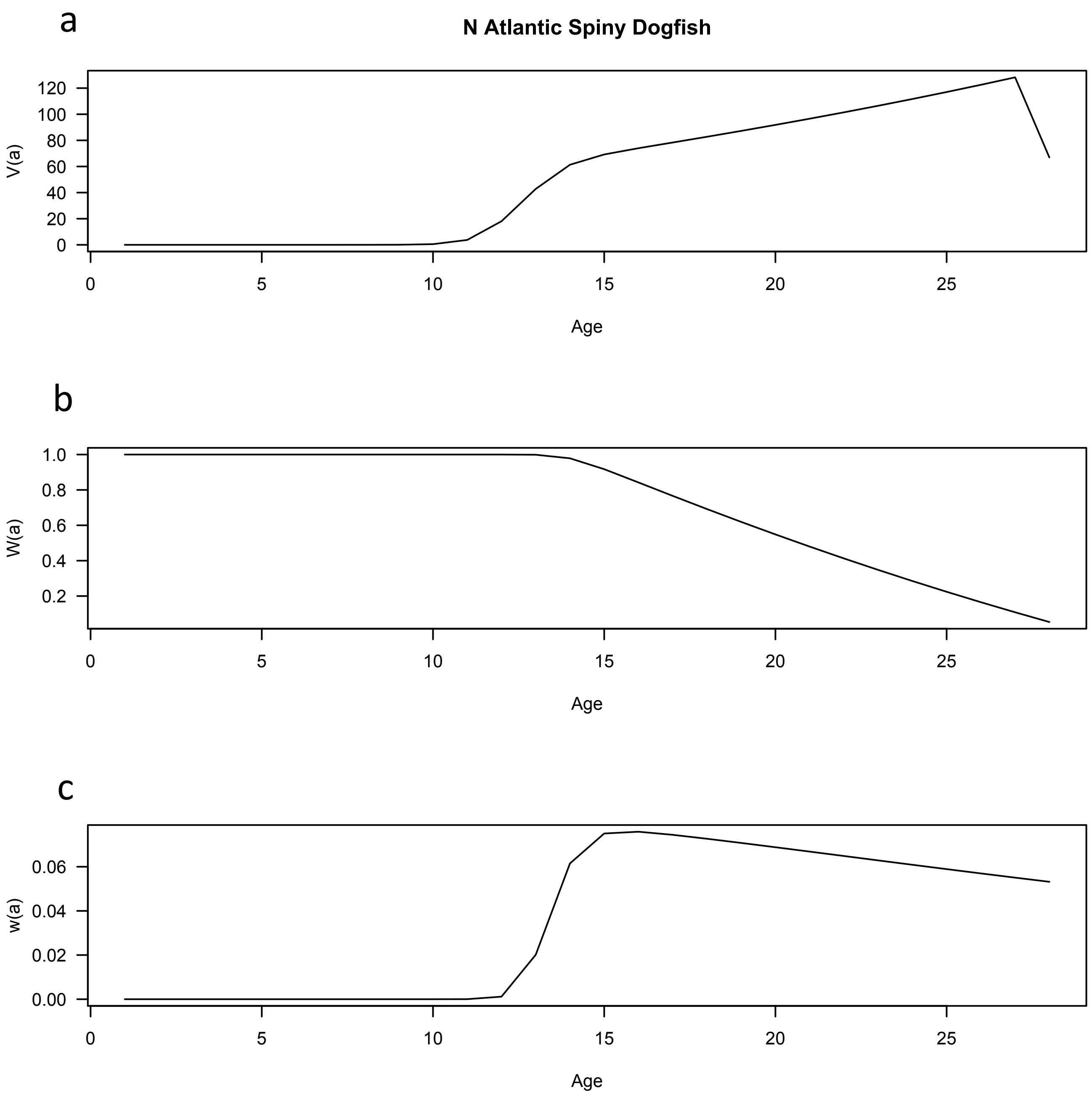

Figure S3.2: N. Atlantic Spiny Dogfish (*Squalus acanthias)*

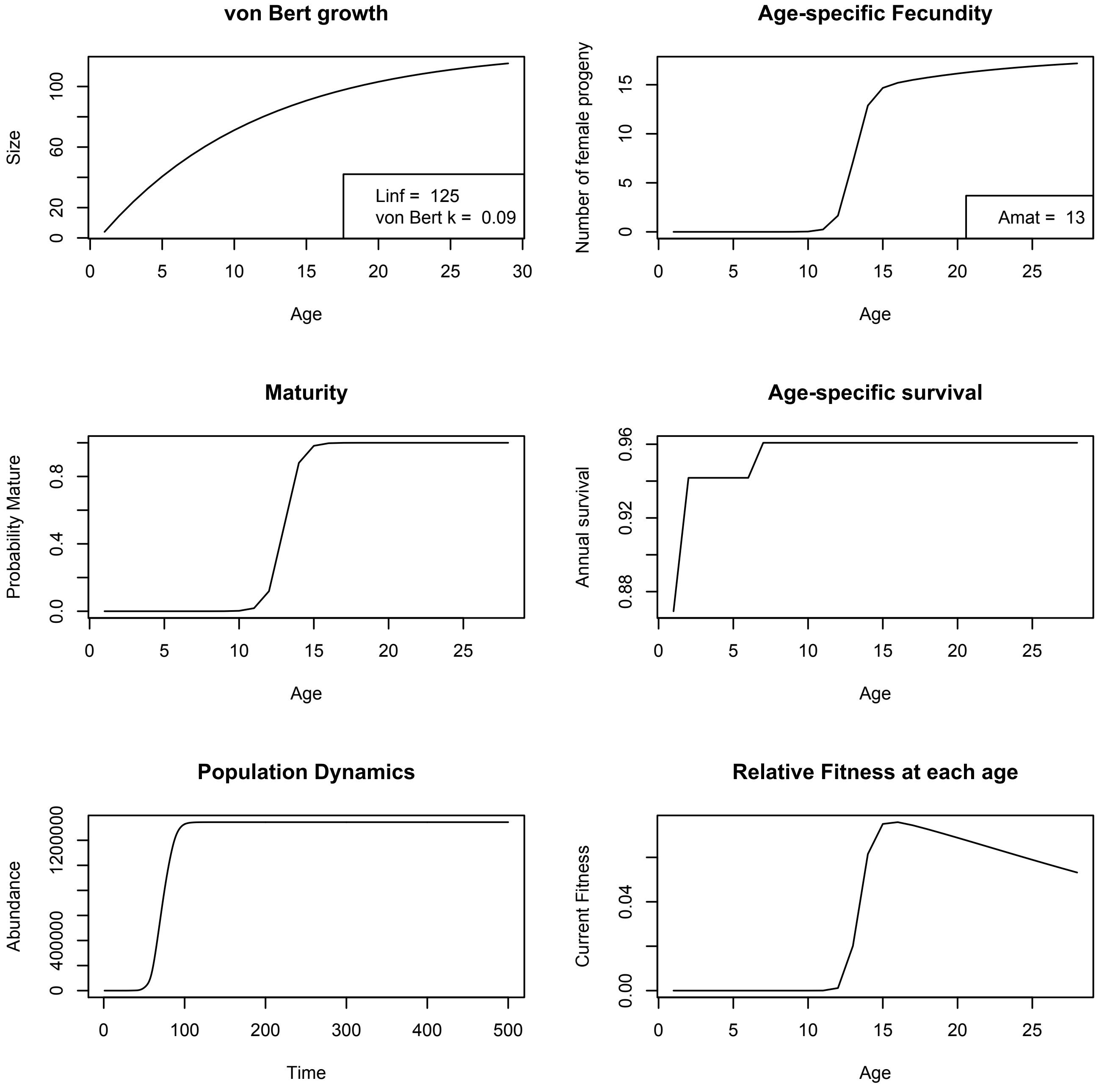

Figure S3.3: Winter Skate (*Leucoraja ocellata*)

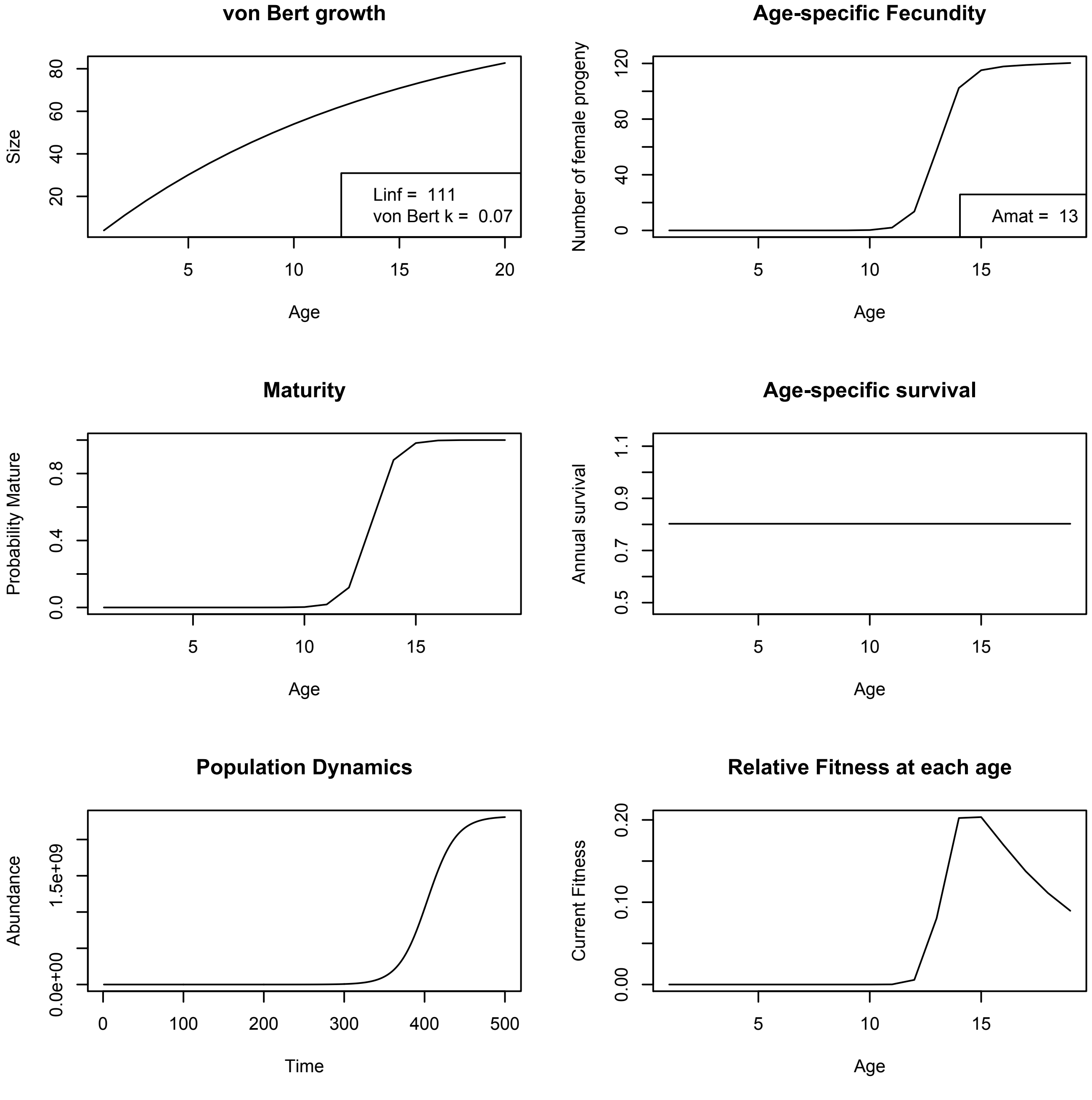

Figure S3.4: North Pacific Bluefin Tuna (*Thunnus orientalis*)

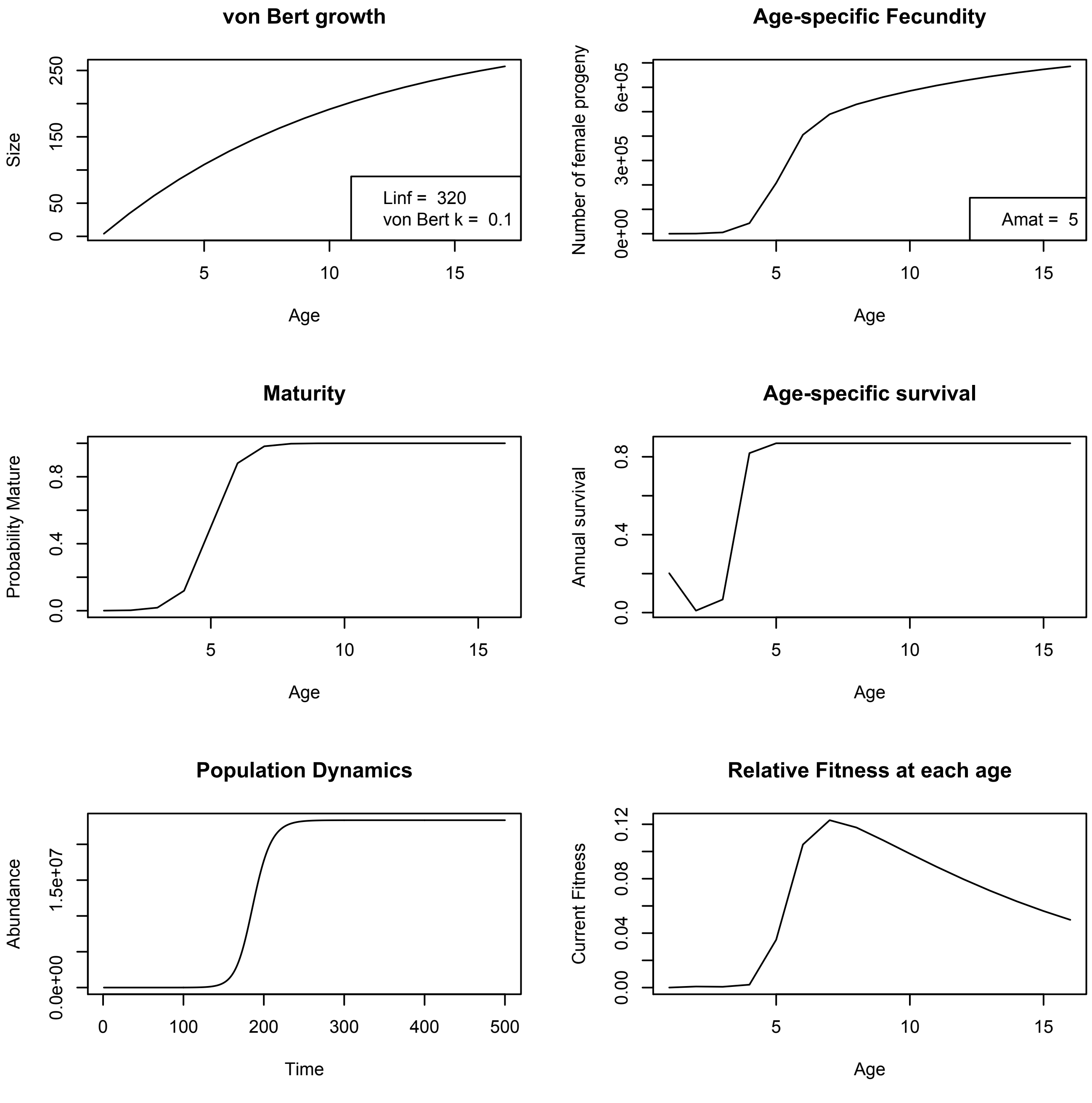

Figure S3.5: Yellowfin Tuna (*Thunnus albacares*)

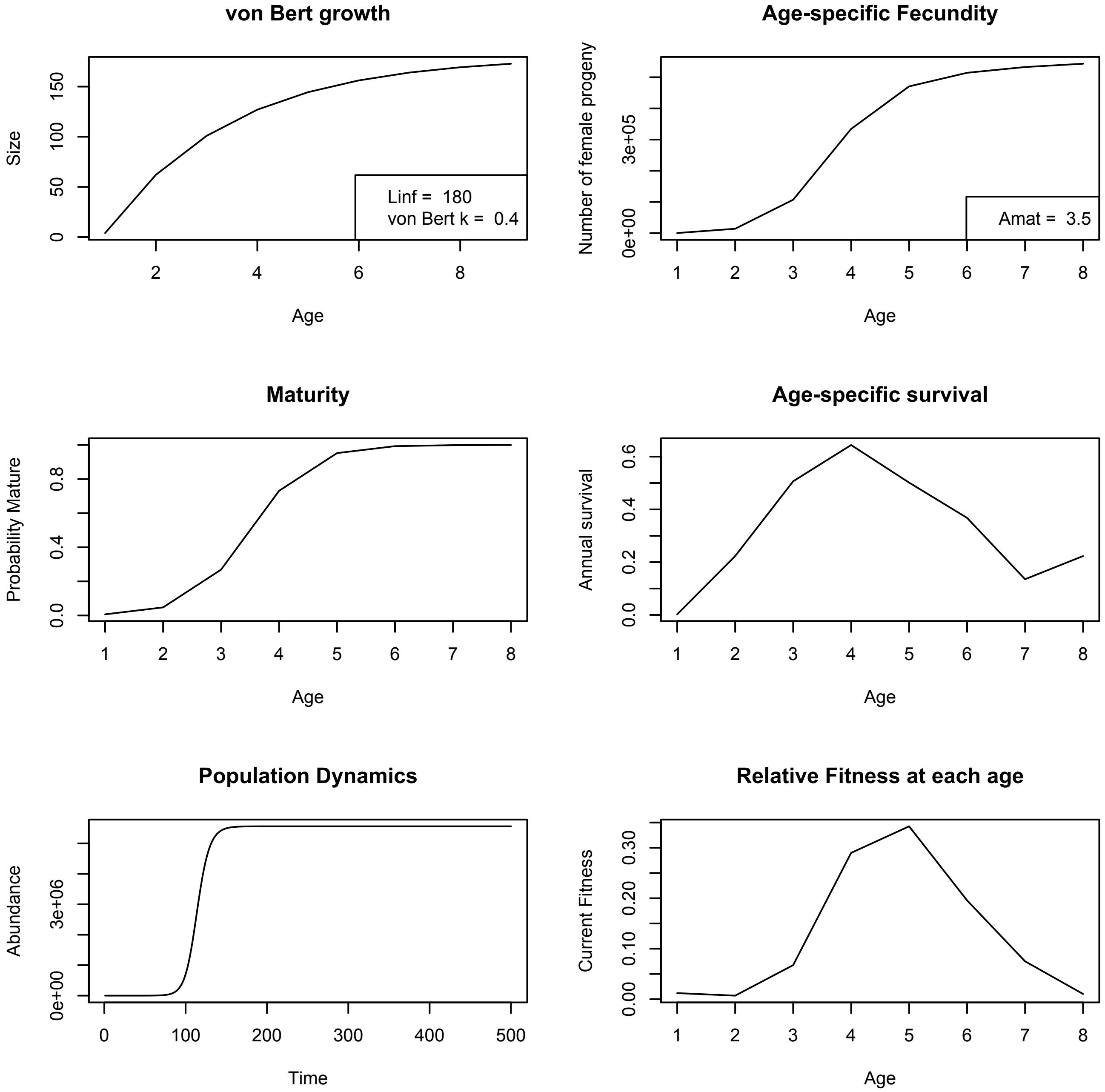

Supplement: Supplementary file 3 — Appendix S3. Calculating reproductive value. Table S3.1. Data used to calculate relative fitness of each age in Box 2. Fig. S3.1. In (a) we plot V(a) over age using Eq. S3.1 and estimates of age‐specific mortality, maturity and length. [file ECE3-6-2125-s003.pdf]
